# Tailoring Cu–SiO_2_ Interaction through Nanocatalyst Architecture to Assemble Surface Sites for Furfural Aqueous-Phase Hydrogenation to Cycloketones

**DOI:** 10.1021/acsami.4c05266

**Published:** 2024-07-30

**Authors:** Welington L. S. Soares, Leon F. Feitosa, Carla R. Moreira, Francine Bertella, Christian Wittee Lopes, Andréa M. Duarte de Farias, Marco A. Fraga

**Affiliations:** † Instituto Militar de Engenharia, Praça Gen. Tibúrcio 80, Urca, Rio de Janeiro, Rio de Janeiro 22290-270, Brazil; ‡ Laboratório de Catálise, Instituto Nacional de Tecnologia—INT, Avenida Venezuela, 82/518, Saúde, Rio de Janeiro, Rio de Janeiro 20081-312, Brazil; § Departamento de Química, Universidade Federal do Paraná (UFPR), Curitiba, Paraná 81531-990, Brazil

**Keywords:** core−shell, nanostructured catalyst, furfuryl alcohol, biomass, green chemistry, biorefinery

## Abstract

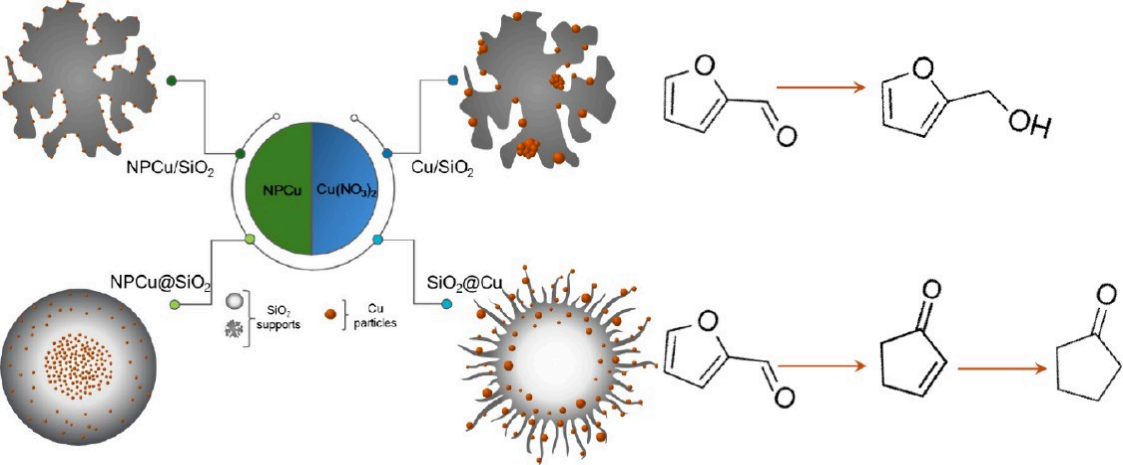

In this contribution, nanocatalysts with rather diverse architectures were designed to promote different intimacy degrees between Cu and SiO_2_ and consequently tune distinct Cu–SiO_2_ interactions. Previously synthesized copper nanoparticles were deposited onto SiO_2_ (NPCu/SiO_2_) in contrast to ordinarily prepared supported Cu/SiO_2_. NPCu@SiO_2_ and SiO_2_@Cu core–shell nanocatalysts were also synthesized, and they were all bulk and surface characterized by XRD, TGA, TEM/HRTEM, H_2_-TPR, XANES, and XPS. It was found that Cu^0^ is the main copper phase in NPCu/SiO_2_ while Cu^2+^ rules the ordinary Cu/SiO_2_ catalyst, and Cu^0^ and electron-deficient Cu^δ+^ species coexist in the core–shell nanocatalysts as a consequence of a deeper metal–support interaction. Catalytic performance could not be associated with the physical properties of the nanocatalysts derived from their architectures but was associated with the more refined chemical characteristics tuned by their design. Cu/SiO_2_ and NPCu/SiO_2_ catalysts led to the formation of furfuryl alcohol, evidencing that catalysts holding weak or no metal–support interaction have no significant impact on product distribution even in the aqueous phase. The establishment of such interactions through advanced catalyst architecture, allowing the formation of electron-deficient Cu^δ+^ moieties, particularly Cu^2+^ and Cu^+^ as unveiled by spectroscopic investigations, is critical to promoting the hydrogenation–ring rearrangement cascade mechanism leading to cycloketones.

## Introduction

The current climate emergency scenario we have been experiencing is a consequence of the long-term use of fossil resources on which the 20th century economy was established. Transitioning to a bioeconomy relying on renewable resources, waste materials, and sustainable and low-carbon technologies is an urge. Exploiting biomass as feedstock has thus been a strategy, particularly the waste biomass made available in well-established commercial processes. Lignocellulose definitely holds the prime role in this scenario as it is widely available, cheap, and composed of interesting chemical structures within its carbohydrate and lignin fractions. The monosaccharides, furan, and aromatics derived thereof can be taken to produce chemicals, biofuels, and other biofuel-related products.^1−3^

Furfural is among the most promising and versatile biomass-derived molecules as it can lead to dozens of other chemicals due to its reactivity with an aldehyde group attached to a furan ring. Mastering its conversion to crucial bioproducts is very challenging though, and as far as the chemical industry is concerned, the development of suitable and very selective catalysts is the major hurdle. Hydrogenation of furfural has been extensively studied as furfuryl alcohol, tetrahydrofurfuryl alcohol (THFA), 2-methyltetrahydrofuran (2-MTHF), 2-methylfuran, furan, tetrahydrofuran (THF), cyclopentenone, cyclopentanone, and cyclopentanol can be formed. Furfuryl alcohol is by far the most intended one, as around 60% of furfural produced worldwide is forwarded to its industrial production. Liquid-phase hydrogenation of furfural to furfuryl alcohol involves the dissociative chemisorption of hydrogen and the adsorption of furfural on a metal center in a way it promotes the activation of C=O bond while protecting the furan ring.^4−6^ Cu–Cr catalysts are the preferred ones, but a wide variety of catalytic systems, including noble metal based catalysts, have been investigated over the years due to the environmental and health issues related to the use and handling of chromium.^4,7^ Production of cycloketones, on the other hand, has been drawing increasing attention due to their use in the syntheses of antifungal agents, remedies, flavors, and fragrances,^8^ reaching the specialty chemicals market, and in the production of linear or singly branched hydrocarbons for diesel and aviation fuel applications.^9^ Furthermore, conventional reaction routes to obtain cyclopentanones are all based on fossil resources,^10−12^ and therefore an alternative, green, and more sustainable process is highly demanded as recently reviewed.^13^

Since the first work on cyclopentanone production from furfural over Pt, Pd, and Ru supported catalysts,^14^ other studies kept exploiting the behaviors of those noble metals^15−18^ extending the investigations to Au^18,19^ and Pt and Pd bimetallic catalysts for which Cu,^17,20^ Fe,^17,18^ and Cr^18^ were used as promoters. Non-noble Co,^20−24^ Fe,^25^ Cu,^26−30^ and Ni^17,31−34^ metal catalysts as well as bimetallic systems made thereof^21,25,28,35,36^ have also been evaluated. Despite the widespread previous use of Cu-based catalysts for furfural hydrogenation, furfuryl alcohol is usually the major product and chemoselectivity competes with the formation of tetrahydrofurfuryl alcohol and 2-methylfuran.^37−41^ The presence of water is crucial to drive the reaction to cycloketones as first reported by Hronec et al.,^14^ disclosing that the reaction intermediate in the furan ring rearrangement is electrophilic in nature. Lewis acid sites are claimed to play a role in the reaction as they would promote the dehydration of 4-hydroxy-2-cyclopentenone, which is formed when protonated furfuryl alcohol interacts with water for ring opening, leading to 2-cyclopentenone that may be hydrogenated to cyclopentanone and cyclopentanol.^42^ A weak acidity requirement could be foreseen from the early nonacidic carbon-supported catalysts used by Hronec et al.^14−16,20^ to several other systems supported on nonacidic or weak acidic materials such as SiO_2_,^29,35^ carbon nanotubes^30,33^ and hollow spheres,^43^ and Al_2_O_3_, TiO_2_, and ZrO_2_.^22,29^ However, some controversial results were reported as Cu supported on SiO_2_^41^ or Al_2_O_3_, Al_2_O_3_–ZrO_2_, Al_2_O_3_–TiO_2_, and Al_2_O_3_–MgO^40^ provided only furfuryl alcohol and 2-methylfuran even when water was used as solvent. Likewise, more strongly acidic supports such as solid acid resins^34^ and Nb_2_O_5_^23^ have been more recently claimed to be required to lead to cycloketones.

This work aims at contributing to this open discussion by investigating the performance of Cu–SiO_2_ catalysts in the aqueous-phase hydrogenation of furfural to cycloketones. Cu was chosen due to its low cost and high availability^42,44^ as well as its ability to reduce carbonyl bonds rather than participating in saturation of C=C ring bonds in the furfural molecule. Moreover, SiO_2_ is expected to be an inert support with no relevant interaction with the substrate to drive the reaction pathway by itself. In the present approach, nanocatalysts with rather diverse architectures were designed to promote different intimacy degrees between Cu and SiO_2_ and consequently tune distinct Cu–SiO_2_ interactions.

## Materials and Methods

### Catalyst Synthesis

Initially, copper nanoparticles (NPCu) were synthesized in diethylene glycol, using CuSO_4_·5H_2_O as metal precursor (0.2 mol/L), NaH_2_PO_2_ as reducing agent (0.6 mol/L), and polyvinylpyrrolidone (PVP, 4 wt %) and cetyltrimethylammonium bromide (CTAB, 0.1 mol/L) as stabilizing agents. Solutions containing PVP, CTAB, and NaH_2_PO_2_ were mixed at 100 °C. It was then heated to 140 °C, and finally the copper precursor solution was slowly added to it and was kept stirring for 5 min, whereafter it was cooled to room temperature.^45,46^ This final colloidal suspension was diluted in water and mixed with a powder silica (53–63 μm) supplied by Saint Gobain, keeping a mass/solid ratio of 1/7. In a typical synthesis procedure, a 6 mL aliquot of colloidal NPCu suspension was diluted in 30 mL of water and used to disperse 5 g of SiO_2_. This mixture was stirred for 3 h at room temperature, the solvent was removed under vacuum at 60 °C, and the obtained powder was finally dried in an oven at 110 °C for 12 h. This nanocatalyst was named NPCu/SiO_2_.

The same NPCu colloidal suspension was used to synthesize the NPCu@SiO_2_ core–shell nanocatalyst. A 0.06 g sample of NPCu was added to a previously prepared solution containing 7 mL of NH_4_OH, 104 mL of ethanol, and 83 mL of water. It was magnetically stirred while 0.3 g of CTAB and lastly 1 mL of tetraethyl orthosilicate (TEOS) were added. The solution was kept stirring for 24 h at room temperature, and the powder was collected by centrifugation. It was extensively washed, dried at 60 °C for 3 h in an oven, and finally calcined under a synthetic air flow (30 mL/min) at 500 °C (10 °C/min).^47^

To obtain a SiO_2_@Cu nanocatalyst, SiO_2_ microspheres with a mean size diameter of 300 μm were first synthesized according to the Stöber method. A solution prepared with 100 mL of ethanol and 12.3 mL of TEOS was slowly mixed (1 mL/min) with a second solution containing 100 mL of ethanol, 2.5 mL of water, and 25 mL of NH_4_OH. After, the final solution was kept stirring at 1500 rpm for 1 h at 55 °C. The white powder was centrifuged, washed with water until constant pH, and dried at 100 °C for 12 h. A 1.3 g sample of the as-synthesized SiO_2_ microspheres were suspended in water (70 mL) under magnetic stirring for 20 min. An aqueous solution (80 mL) containing 0.46 g of Cu(NO_3_)_2_·3H_2_O and 8 mL of NH_4_OH was then added and left to age for 25 min under agitation. After that, the suspension was transferred to an autoclave and thermally treated at 140 °C for 12 h. The powder was then separated by centrifugation, washed with water and ethanol, and dried at 80 °C for 18 h.

Ordinary Cu/SiO_2_ catalyst was prepared by incipient wetness impregnation using Cu(NO_3_)_2_·3H_2_O as metal precursor. After impregnation, the catalyst was dried in an oven at 100 °C for 18 h and calcined at 400 °C (10 °C/min) under synthetic air (30 mL/min) for 2 h.

### Catalyst Characterization

X-ray diffraction (XRD) was used to investigate the existence of crystalline phases in the synthesized catalysts. All analyses were performed in Bruker D8 Advance equipment using a Cu Kα radiation source and operating at 40 kV and 40 mA. The diffractograms were collected in the 10–90° 2θ Bragg angle region, with a 0.02°/step increment and a 0.5 s/step counting time. Software EVA and COD database were used to identify the crystalline phases present in each sample. Crystallite domain average sizes were calculated from the CuO(002) plane (2θ = 35.5°) line by the application of the Scherrer equation.

The presence of organic material reactants in the synthesized catalysts was investigated by thermogravimetric analysis (TGA). The analyses were performed in SDT Q600 equipment (TA Instruments). Approximately 10 mg was placed in the sample cup and submitted to a 20 °C/min heating rate from room temperature up to 800 °C under oxidizing atmosphere (synthetic air, 100 mL/min).

Catalyst morphologies were investigated by a field emission gun scanning electron microscope (FE-SEM), an FEI Helios Nanolab Dual Beam G3 CX equipped with a transmission mode detector. The scanning transmission electron mode on an SEM microscope (STEM-in-SEM) operates at 30 kV and includes a specific sample holder for conventional copper TEM grids and a high-angle annular dark-field STEM detector (HAADF-STEM). Despite the lower resolution compared to conventional TEM at typical voltages (80–200 kV), the STEM-in-SEM offers a contrast enhancement and avoids chromatic aberrations. (HR)TEM images were recorded using a 200 kV 2100F Jeol instrument. Complementary TEM analyses were performed in a 200 kV Talos F200X Thermo Fisher microscope. The elemental mapping images of the samples were obtained with an energy dispersive X-ray detector (EDX) coupled to the TEM.

Samples were previously suspended in ethanol and dispersed ultrasonically for 15 min. A drop of the suspension was deposited on a copper grid coated with a holey carbon film. To elucidate inside different nanoarchitectures of the catalysts, some samples were embedded in Spurr’s resin at ambient temperature followed by polymerization at 80 °C for a period of 12 h. Finally, ultrathin sections (range 50–80 nm) were cut with an ultramicrotome (PT-PC/RMC Boeckeler) using a diamond knife and placed on a holey carbon coated copper TEM grid.

Interplanar distances of the phases found in the catalysts were determined by fast Fourier transform (FFT) analysis of the HRTEM micrographs using Gatan’s Digital Micrograph software version 3. Particle size measurements were performed using Image-J (1.52a). More than 100 particles were taken for the statistical purpose.

With the use of Autochem 2920 equipment (Micromeritics) with a thermal conductivity detector (TCD), the Cu-based catalysts were analyzed by temperature-programmed reduction (H_2_-TPR). A 100 mg sample of catalyst was pretreated at 130 °C, under N_2_ (50 mL/min), for 30 min. After cooling of the sample to room temperature, the reduction was conducted from 60 to 700 °C, under the gas mixture H_2_/N_2_ (10 vol % H_2_).

X-ray absorption near-edge structure (XANES) analyses were carried out on the Carnaúba beamline of Sirius, the Brazilian synchrotron light source at the National Center for Research in Energy and Materials (CNPEM). A beam size of approximately 400 nm × 800 nm was used, with energy corresponding to 9725 eV, sufficient to excite the electrons on the K edge of Cu. The spectra were collected in the interval of −20 to +40 eV with respect to the *E*_0_ value of Cu, using 0.5 eV of energy resolution. The reduction of data was performed using the IFEFFIT package.^48^

X-ray photoelectron spectroscopy (XPS) was used to investigate Cu species present on the catalyst’s surface. The analyses were performed in a hemispherical energy analyzer (Specs Phoibos 150) equipped with an Al Kα (1486.6 eV) nonmonochromatic source. The equipment base pressure during analysis was less than 10^–10^ mbar. Spectra were recorded in two different conditions. Survey spectra were obtained using a 50 eV pass energy, 1.0 eV step, and 15 scans. High-resolution spectra (Cu 2p, Cu LMM, C 1s, Si 2p, and O 1s regions) were generated employing a 20 eV pass energy, 0.08 eV step, and at least 25 scans. The C 1s adventitious carbon binding energy (BE) at 286.6 eV was used as reference for spectral calibration. CasaXPS software version 2.3.15 (Casa Software Ltd., Cheshire, U.K.) was used to treat the obtained data. Spectral components were determined by fitting procedures using Tougaard (Cu 2p) and Shirley (survey and other high-resolution regions) baselines. Gaussian (70%)–Lorentzian (30%) functions were used to represent Cu^2+^ and satellite components, and Gaussian (10%)–Lorentzian (90%) functions were used for Cu^+^/Cu^0^ components.

Inductively coupled plasma optical emission spectrometry (ICP-OES) was used to determine the experimental Cu loadings of the synthesized catalysts and to evaluate Cu leaching in the catalytic tests. To quantify the Cu content of each fresh catalyst, liquid samples were prepared by adding 25 mg of the solid sample to an acid solution (3 mL of HF + 1.5 mL of HCl + 1 mL of HNO_3_), mixing it with 10 mL of *aqua regia*, keeping this solution under heating (100 °C/15 min), and finally diluting it in a 100 mL volumetric flask with a 1% HNO_3_/H_2_O solution. In the case of Cu leaching evaluation, the liquid samples consisted of filtered (45 μm) postreaction media. All those samples were analyzed with a Varian Vista MPX spectrometer.

The textural properties of the synthesized materials were evaluated by nitrogen physisorption at −196 °C using Micromeritics ASAP 2040 equipment. Prior to analysis, the samples were submitted to outgassing under vacuum at 150 °C. The obtained data were treated with the BET (Brunauer–Emmett–Teller) equation in order to calculate the materials’ total surface area. Moreover, BJH (Barrett–Joyner–Halenda) formalism was used to estimate the materials’ pore volume and average pore size from the isotherm desorption branch.

### Catalytic Evaluation and Product Analysis

The nanocatalysts were tested in the aqueous-phase hydrogenation of furfural in a 10 mL batch reactor at 150 °C and pressure control of hydrogen at 30 bar. Reaction runs were carried out at different furfural:Cu mass ratios (5:1; 10:1, 20:1) and reaction times (4 and 8 h) starting after the system reached the set temperature. Before reaction, nanocatalysts were activated under hydrogen (10 mL/min) at 300 °C for 2 h. Besides furfural (99%, Sigma-Aldrich), furfuryl alcohol (98%, Sigma-Aldrich) was also used as substrate. They both were used without further purification. After reaction, the reactor was cooled down with the aid of an ice bath and a liquid aliquot was collected and filtered (45 μm) before analysis. Substrate consumption and products formed during reaction were analyzed in an Agilent 7890A flame ionization gas chromatograph (GC-FID) using an HP-Innowax column (0.25 μm × 30 m × 0.25 mm) with helium as carrier gas, a temperature window within 80–180 °C, and a 10:1 split ratio. Products were identified by the chromatographic retention time using standard samples and mass spectrometry fragmentation profile using an Agilent 5975C mass spectrometer detector (GC–MS). Reaction runs were carried out in triplicate, and the results were within an error of ≤5%.

Furfural conversion (*X*), product selectivity (*S*), and carbon balance (CB) were calculated according to eqs 1–3:
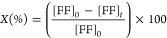
1
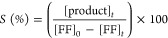
2
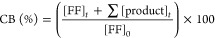
3where [FF]_0_ is the initial concentration of furfural and [FF]_*t*_ and [product]_*t*_ are the concentrations of furfural and products formed at the final time (*t*) reactions were performed.

## Results and Discussion

**Figure 1:**
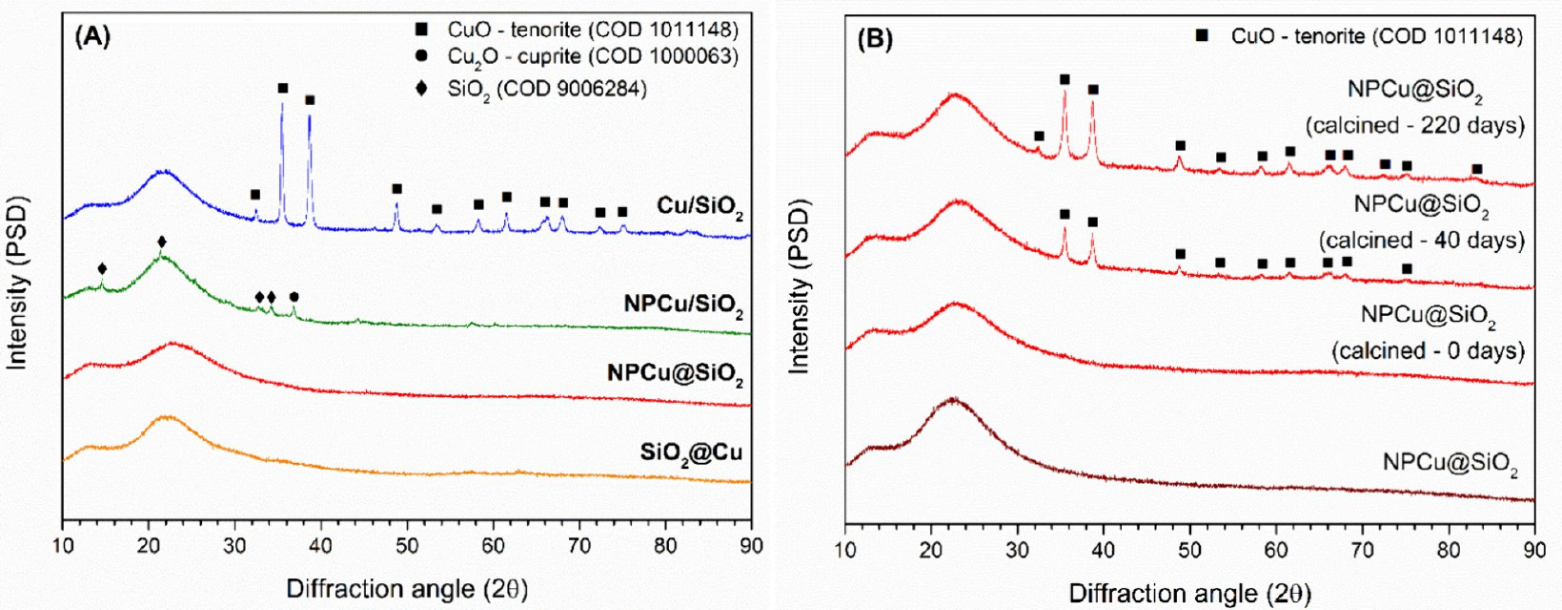
Diffractograms of Cu–SiO_2_ nanocatalysts. Comparison of all synthesized materials (A) and evolution of NPCu oxidation in NPCu@SiO_2_ after calcination (B).

The diffractograms of Cu–SiO_2_ nanocatalysts are presented in Figure 1. All samples exhibit two large halos in the 10–30° range, which are characteristic of noncrystalline SiO_2_ materials (Figure 1A). Moreover, only Cu/SiO_2_ and NPCu/SiO_2_ present additional peaks related to crystalline phases. Cu/SiO_2_ diffraction peaks can all be attributed to the presence of the tenorite (CuO) phase (COD 1011148). The use of the Scherrer equation indicates that the average size of a CuO crystallite in Cu/SiO_2_ is 27 nm, substantiating the nanosized crystalline domains in line with other reports in the literature in which the mean size of CuO varies between 20 and 35 nm for similar preparation procedures.^49−51^ As for the NPCu/SiO_2_ nanocatalyst, no CuO could be detected while a peak centered at 36.8° can be attributed to the cuprite (Cu_2_O) phase (COD 1000063). Additional diffractions at 14.6, 21.4, 32.8, and 34.3° can be associated with SiO_2_ (COD 9006284). The fact that no Cu phase diffraction peaks are present in SiO_2_@Cu and NPCu@SiO_2_ suggests that Cu crystal domain sizes in those nanocatalysts are very small and below the detection limit for ordinary XRD analyses.

Once the same NPCu were used to synthesize NPCu/SiO_2_ and NPCu@SiO_2_, it would be expected that both catalysts would present the same pattern of Cu phases. Moreover, the used procedure to obtain NPCu is expected to generate Cu^0^ nanoparticles.^45,46^ The particular occurrence of a Cu_2_O phase in NPCu/SiO_2_ can be indicative of a partial oxidation of metallic Cu^0^ upon deposition of NPCu onto the SiO_2_ surface during the catalyst preparation. It is conceivable that the PVP used as stabilizing agent in NPCu synthesis is washed out during NPCu/SiO_2_ preparation, leaving NPCu more susceptible to oxidation. However, an eventual agglomeration of Cu_2_O phase during deposition on SiO_2_, leading to the rise of its diffraction peak, cannot be ruled out. In the case of the as-synthesized NPCu@SiO_2_ core–shell nanomaterial, on the other hand, the NPCu particles are covered and protected by the SiO_2_ shell as well as by the residual PVP left after NPCu washing, and the CTAB surfactant used to assist the silica shell formation, being more resistant to oxidation and agglomeration. It is indeed important to recall that, before the synthesis of both NPCu/SiO_2_ and NPCu@SiO_2_, the NPCu were washed only to remove the excess of CTAB and PVP.

**Figure 2:**
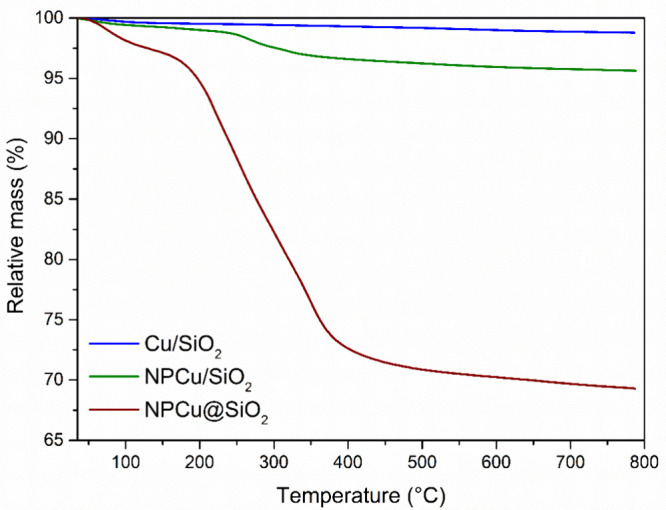
Thermogravimetric curves of Cu/SiO_2_, NPCu/SiO_2_, and NPCu@SiO_2_ nanocatalysts obtained in oxidizing atmosphere.

The presence of the remaining organic matter is confirmed by TGA profiles obtained in oxidizing atmosphere displayed in Figure 2 for those two nanocatalysts. The thermogram of Cu/SiO_2_ is also shown as a reference of a catalyst that does not require any organic reactants during preparation. The more or less noticeable mass loss event registered below 150 °C is attributed to the loss of water molecules. Nevertheless, a more clearly pronounced event occurs within the 150–800 °C temperature range.

The mass losses for NPCu/SiO_2_ (3.61%) and mainly for NPCu@SiO_2_ (27.82%) are much more relevant if compared to the ordinary Cu/SiO_2_ sample (0.79%) and are associated with the organic materials’ burn off in the as-synthesized nanocatalysts, which is indeed in a much higher amount in NPCu@SiO_2_ due to the use of CTAB in addition to PVP. TGA relative mass derivative curves of these organic compounds (Figure S1) corroborate this conclusion as CTAB is oxidized between 200 and 300 °C and PVP is oxidized between 300 and 650 °C.

Further evidence of the role played by the capping agent in avoiding oxidation of NPCu in NPCu@SiO_2_ is provided by the long-term (220 days) monitoring of crystalline phases by XRD after calcination to remove all stabilizer and surfactant (Figure 1B). As-synthesized and just-calcined NPCu@SiO_2_ nanocatalyst presents no diffraction peaks related to any Cu phase, nor metallic Cu^0^ as already discussed. However, after calcination and storage, CuO peaks clearly appear and their intensity increases through time, which is associated with an increase in the fraction of such an oxidized Cu phase upon air exposure.

**Figure 3:**
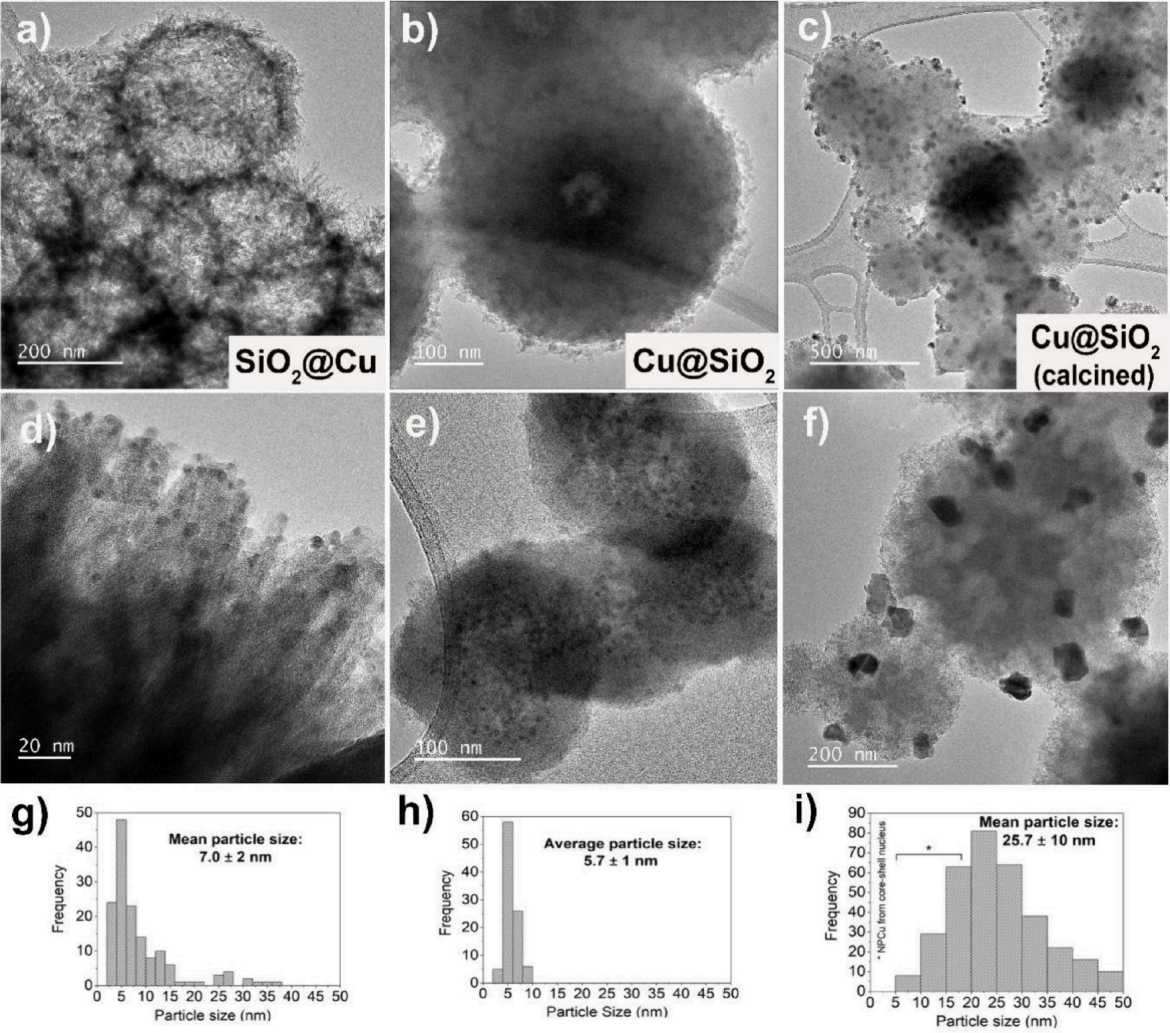
TEM micrographs of catalysts and particle size distribution: (a, d, g) SiO_2_@Cu, (b, e, h) NPCu@SiO_2_, and (c, f, i) NPCu@SiO_2_ after calcination.

Morphology, nanoparticle size, and identification of Cu phases at the nanoscale were assessed by TEM and high-resolution transmission electron microscopy (HRTEM). TEM micrographs and particle size distributions of all nanocatalysts are presented in Figures 3 and S2. Despite the average size of copper nanoparticles of around 27 nm as estimated from the Scherrer equation using the XRD pattern, TEM examination of the Cu/SiO_2_ sample (Figure S2a) discloses it also contains small copper nanoparticles with an average size of 2.6 nm (Figure S2d), in a well-dispersed arrangement over the silica support. Figure S2b displays the colloidal NPCu directly dispersed on the grid and the estimated nanoparticles’ average size is 3.1 nm (Figure S2e). In addition to the well-formed Cu nanoparticles, the presence of organic material used in the synthesis is also easily noticeable in the image. SEM and STEM-HAADF analyses (Figure S3a,b) further corroborate it. Figure S2c, collected from the NPCu/SiO_2_ nanocatalyst, reveals that the dispersion procedure applied herein does not bring any change to the nanoparticles themselves, which now exhibit a mean diameter size of around 3.6 nm (Figure S2f).

**Figure 4:**
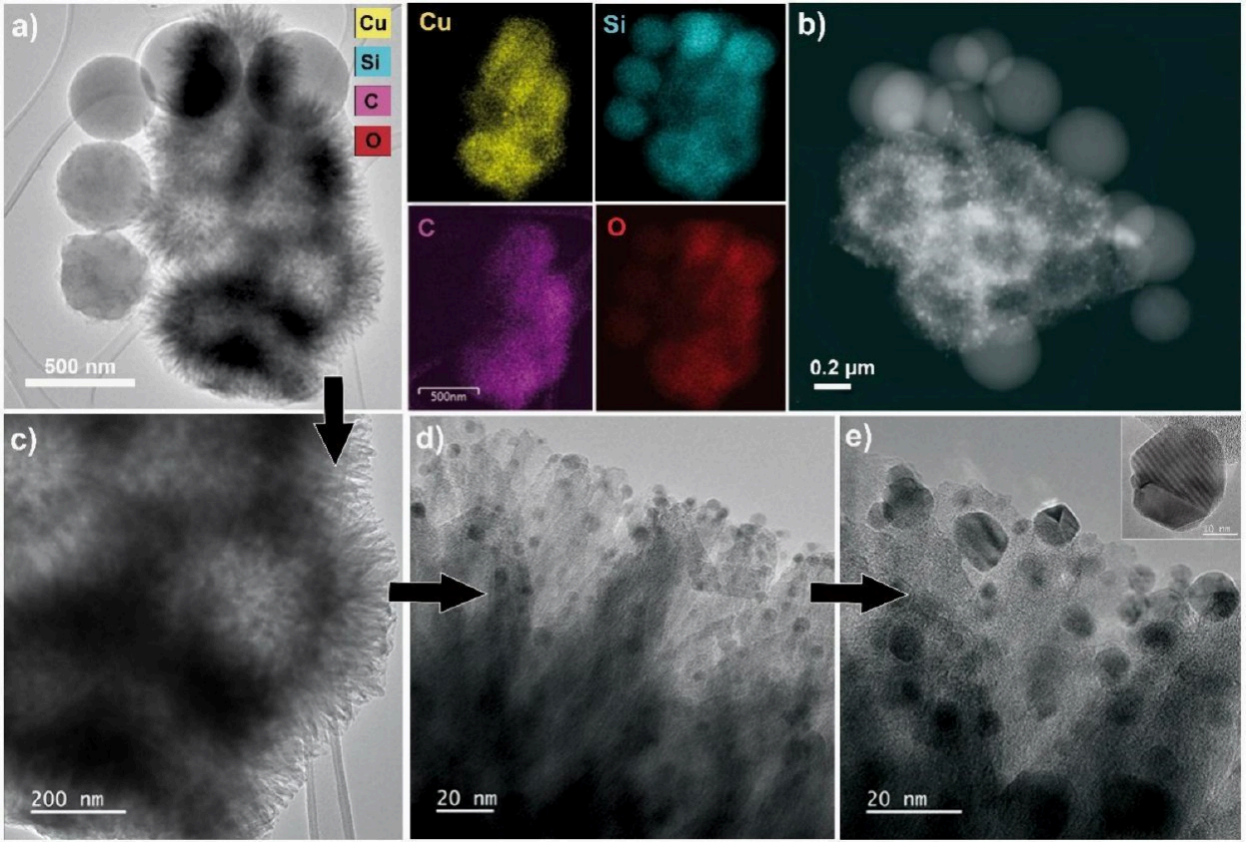
(a) TEM and (b) STEM-HAADF micrographs of SiO_2_@Cu catalyst. Higher magnification micrographs (c, d, e) of the spikes around SiO_2_ spheres, exhibiting NPCu at the tips. EDX elemental mapping of (a) micrograph: Si, cyan; Cu, yellow; C, magenta; and O, red. Cu nanoparticles HRTEM image in the inset (e).

TEM micrographs of SiO_2_@Cu nanocatalyst are shown in Figure 3a,d, exhibiting urchin-like SiO_2_ spheres that are surrounded by different sizes of amorphous spikes (Figures 3d and 4a,c). Copper nanoparticles are homogeneously dispersed over the SiO_2_ amorphous spikes (Figures 3d and 4d), with a higher mean size (7.0 nm) when compared to the previously described nanomaterials. STEM-HAADF analysis (Figure 4b) confirms the presence of copper nanoparticles because of their brightness (Z contrast), allowing seeing shiny dots throughout the urchin-like structure. The EDX elemental mapping (Figure 4a) shows that Si, O, Cu, and C elements are well dispersed, which could favor the establishment of a strong interaction between Cu and the SiO_2_ support.

Figure 4a,b also displays some isolated SiO_2_ spheres. The high-resolution TEM image (inset in Figure 4e) and its corresponding fast Fourier transform (FFT) did not generate sufficient assertive data to distinguish the possible Cu phases, once the lattice distances are too close to each other, but they endorse the high crystallinity of the copper nanoparticles over the silica tips. The collected interplanar distances (*d* = 2.50 Å and *d* = 2.10 Å) are more frequently fit as Cu(I). However, 1.60 and 2.0 Å were also found as interplanar distances for this sample, indicating the presence of Cu^0^ metallic copper.

**Figure 5:**
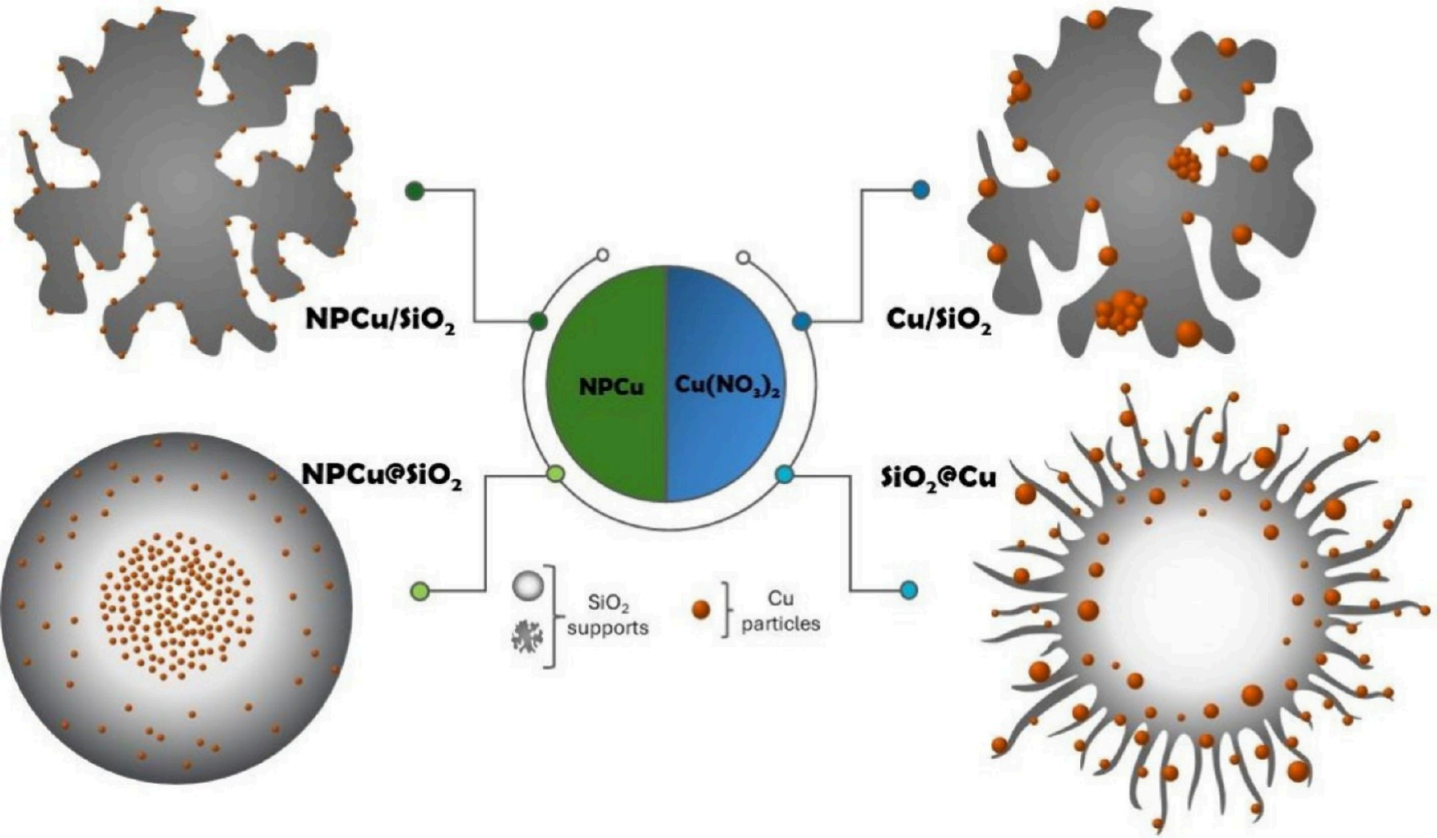
Schematic representation of the nanoarchitectures of the synthesized catalysts according to TEM analysis.

Encapsulation of copper nanoparticles rendered the NPCu@SiO_2_ core–shell nanocatalyst in which NPCu are embedded within a silica thicker sphere (about 200 nm) inside a smaller cavity (Figures 3b and S4a,b) and dispersed inside thinner hollow SiO_2_ spheres (thickness < 60 nm) (Figures 3e and S4c). In the thicker sphere, it is possible to see an interface of about 25–50 nm between the silica shell and the hollow small nucleus that houses the copper nanoparticles and contains both Si and Cu, with a higher concentration of Cu nanoparticles (darker round region in Figures 3b and S4b; bright round region in Figure S4a). The encapsulation of NPCu holds the grain growth; however, the NPCu outside the nucleus of NPCu@SiO_2_ exhibit a larger average metal diameter of around 5.7 nm (Figures 3e and S4c). EDX elemental mapping (Figure S4a) confirms the core–shell architecture, showing the concentration of Cu (yellow) inside the SiO_2_ shell. The STEM-HAADF result (Figure S4a) shows sparkly points also outside the nucleus, trapped in the thickness of the SiO_2_ shell wall, further confirming the presence of NPCu outside the core. These particles localized in the space between the nucleus and the shell can be clearly observed in Figure S4c. This architecture also favors the Cu–SiO_2_ interaction in a deeper way than those achieved by supporting or depositing copper nanoparticles onto a silica surface. Figure 5 illustrates the NPCu location in all Cu–SiO_2_ catalysts according to TEM results.

The impact of removing the remaining organic matter from the NPCu@SiO_2_ nanocatalyst on copper nanoparticles was also assessed by TEM. Calcination indeed provokes some changes in the catalyst morphology, as larger NPCu can now be seen on the outside surface of SiO_2_ spheres as well (Figure 3c,f), with an average particle size of 25.7 nm. EDX mapping also confirms the presence of larger copper nanoparticles over the SiO_2_ sphere’s surface (Figure S5). The presence of larger pores (in comparison with the as-synthesized nanomaterial) in the SiO_2_ spheres is also easily noticed (Figures 3f and S5). It is suggested that the migration of particles to the surface may cause this enlargement.

Toward a better understanding of the configuration of the inner part of the SiO_2_ shell, the calcined NPCu@SiO_2_ nanocatalyst was embedded in an epoxy resin and sectioned with an ultramicrotome. The resulting lamellas were analyzed by TEM (Figure S6), corroborating that the core–shell structure is well-preserved after calcination, as indicated by the red arrows pointing to the NPCu inside the core (Figure S6a,b); i.e., the NPCu inside the core persist even after calcination and mechanical sectioning. Furthermore, it is evidenced that the NPCu in the hollow SiO_2_ structures (between the core and the shell) agglomerate upon calcination and migrate to the SiO_2_ surface. These nanoparticles are indicated with green arrows in Figure S6a, while the larger pores of the SiO_2_ spheres are signaled by blue ones.

**Figure 6:**
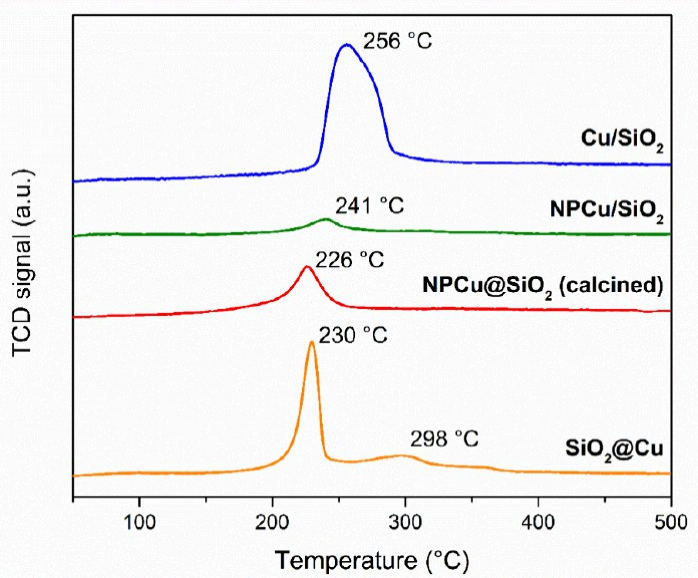
Temperature-programmed-reduction profiles of all nanocatalysts.

The bulk copper phases identified by HRTEM examination were also assessed by investigating their reducibility through H_2_-TPR, and the reduction profiles of the nanostructured catalysts are displayed in Figure 6. All samples reveal reduction peaks, but they notably differ regarding intensity, reduction peak position (reduction temperature), breadth, and number of reduction events.

The conventionally prepared Cu/SiO_2_ exhibits a large, asymmetrical peak centered at 256 °C attributed to the CuO → Cu^0^ reduction step. This range of temperature has indeed been assigned to the presence of bulky CuO phase, including large clusters.^52,53^ However, a shoulder at higher temperature (∼280 °C) is also noted, suggesting the occurrence of a wider distribution of particle sizes in this catalyst. It is worth pointing out that Cu/SiO_2_ XRD data (Figure 1A) indicated the presence of large CuO crystal domains (27 nm), but Cu particles in the range of ca. 2.7 nm were observed by TEM (Figure S2). These data together indeed confirm that this sample features a wide range of particle sizes in good agreement with the larger reduction peak recorded during H_2_-TPR analysis.

Special attention should be paid to the NPCu/SiO_2_ sample, which has a remarkably smaller, nearly symmetrical reduction peak at 241 °C. As previously mentioned, the NPCu synthesis procedure is expected to generate Cu^0^ nanoparticles,^46,47^ and therefore, no reduction peak should be expected. Nevertheless, before they are deposited onto silica, NPCu are washed to remove the excess of PVP (capping agent), which allows the appearance of a fraction of Cu_2_O phase as disclosed by the small peak identified in the XRD pattern (Figure 1A), despite the remaining amount of PVP in the nanocatalyst as evidenced by TGA (Figure 2). The low H_2_ consumption indicated by the NPCu/SiO_2_ H_2_-TPR profile is thus in line with XRD data and leads to the conclusion that the NPCu/SiO_2_ nanocatalyt holds both Cu^0^ and a smaller amount of oxidized Cu^δ+^ species.

The core–shell NPCu@SiO_2_ nanocatalyst also shows a symmetrical well-resolved reduction temperature in the same range as the above-mentioned samples, with a shift to lower position (226 °C), which may be caused by the smaller and narrower particle size distribution. Interestingly, when compared to the supported samples, the encapsulation with SiO_2_ did not disturb the reduction pattern of the core–shell material, indicating full diffusion through the silica shell and access of hydrogen molecules to the metal core. Recording a reduction peak, on the other hand, means that oxidized Cu^δ+^ species are also present, probably coexisting with metallic copper, as observed for NPCu/SiO_2_. Although both NPCu/SiO_2_ and NPCu@SiO_2_ were prepared using the same NPCu, the amount of organic materials is much higher in NPCu@SiO_2_ (Figure 2) due to the additional use of CTAB in NPCu encapsulation by SiO_2_. Therefore, NPCu@SiO_2_ needs to be submitted to a calcination step before its use, which can explain why its reduction peak is more noticeable.

Finally, sample SiO_2_@Cu presents a different profile with not only one but two reduction events; the lower sharp temperature peak (230 °C) is associated with the same Cu^2+^/Cu^0^ reduction step. A clear smaller peak at 298 °C can tentatively be explained by the formation of copper species holding Cu–O–Si bonds rendering a new and stronger interaction between the metal particle and the support, which is indeed in line with the nanoarchitecture accomplished as previously examined by TEM.

**Figure 7:**
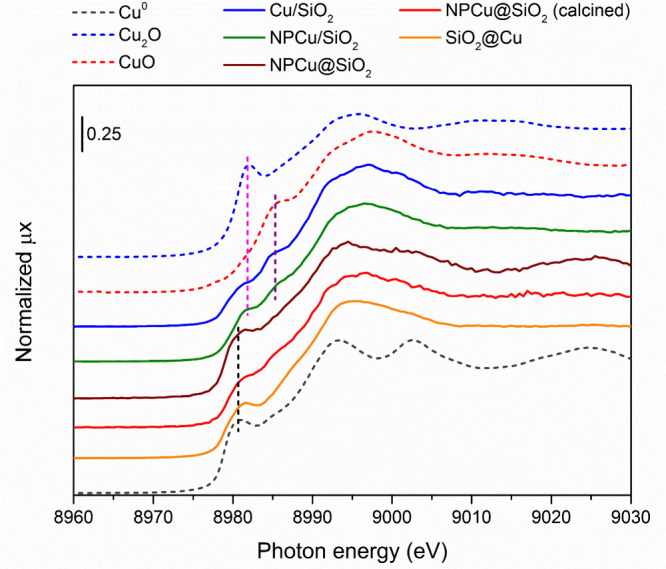
XANES spectra at Cu K edge of as-prepared Cu-based nanocatalysts.

The electronic properties of Cu in the bulk of the as-prepared nanocatalysts was assessed by X-ray absorption near-edge structure (Figure 7). To allow a proper interpretation of the data, a set of reference compounds (CuO, Cu_2_O, and Cu^0^) were used as standards. Once XANES can be considered a fingerprinting technique to investigate oxidation states and local geometry,^54^ it is possible to state that samples show two main spectral profiles: CuO-based and fcc Cu^0^-based profile shapes. Cu/SiO_2_ and NPCu/SiO_2_ spectra majorly coincide with that of CuO reference, in which the shoulder related to 1s → 4p transition of Cu^2+^ is present at ∼8985 eV (purple vertical dashed line).^55^ This information is in good agreement with the observation of the tenorite crystalline phase in Cu/SiO_2_ by XRD. A contribution from reduced copper in these two samples is perceived by the feature at ∼8981 eV. The observation of Cu_2_O in NPCu/SiO_2_ by XRD explains why this sample presents a higher contribution of reduced copper in its XANES (pink vertical dashed line). Attempts to estimate the proportion of reduced copper in Cu/SiO_2_ and NPCu/SiO_2_ samples by linear combination fit between CuO and Cu_2_O did not lead to satisfactory results. The other three samples, namely NPCu@SiO_2_, NPCu@SiO_2_ (calcined), and SiO_2_@Cu, show spectra characteristic of Cu^0^ with an fcc structure,^56^ being those of NPCu@SiO_2_ and SiO_2_@Cu flattened with respect to NPCu@SiO_2_, suggesting the influence of size effects on XANES oscillations (smaller copper domains). Notwithstanding, the NPCu@SiO_2_ spectrum shows a higher whiteline intensity and a perceivable feature within the edge (∼8985 eV), which can be ascribed to a small fraction of Cu^2+^, most likely arising from copper oxidation. This difference between noncalcined and calcined NPCu@SiO_2_ samples indicates that the removal of CTAB and PVP by calcination is followed by a partial oxidation of NPCu.

**Figure 8:**
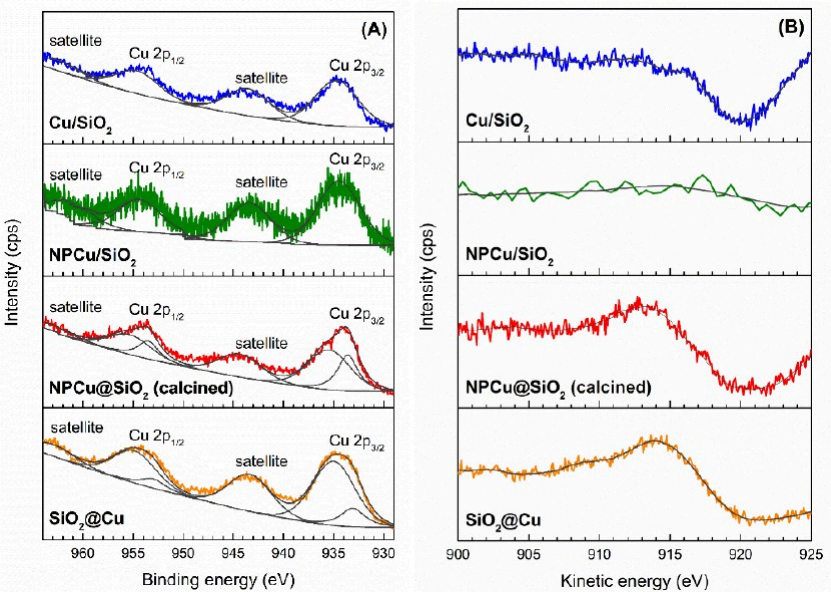
XPS spectra of Cu–SiO_2_ nanocatalysts. (A) Cu 2p region; (B) Cu LMM (Auger) region.

The surfaces of these nanocatalysts were examined by XPS, and the experimental data are summarized in Tables 1 and 2 and Figure 8. Except for the NPCu@SiO_2_ sample, the nanocatalysts are composed of Cu and SiO_2_. Hence, the Si/Cu atomic ratio can be used as indicative of the Cu surface density. Si/Cu values (Table 1) increase in the order SiO_2_@Cu < NPCu/SiO_2_ < NPCu@SiO_2_ < Cu/SiO_2_. According to bulk data obtained from ICP analyses also collected in Table 1, Cu contents in SiO_2_@Cu and calcined NPCu@SiO_2_ are virtually the same and slightly lower than that in Cu/SiO_2_. Therefore, XPS data indicate that different architectures lead to distinct Cu surface densities, and SiO_2_@Cu provides the nanocatalyst with the higher surface metal concentration. It is highlighted that the Cu content was only measured for the calcined NPCu@SiO_2_ sample as the same metal composition can be assumed for its as-synthesized counterpart (NPCu@SiO_2_). Moreover, in relation to NPCu@SiO_2_, only the noncalcined sample consistently presents N on its surface, which disappears after calcination followed by a decrease in C content and an increase in Si and O contents. PVP and CTAB are N-containing organic molecules respectively used in NPCu and NPCu@SiO_2_ syntheses, and this pattern after calcination is in close agreement with previous results from the TGA (Figure 2) and TEM (Figure 3) analyses already discussed.

**Table 1:** Bulk and Surface Compositions of Cu–SiO_2_ Nanocatalysts as Determined by ICP and XPS

		atomic surface composition (%)					
nanocatalyst	bulk Cu content (%)	Cu	Si	O	C	N	Si/Cu
Cu/SiO_2_	9.3	0.17	27.35	40.50	31.98	–	161.9
NPCu/SiO_2_	5.8	0.59	35.12	41.41	22.88	–	59.5
NPCu@SiO_2_	n.a.^a^	0.23	5.12	19.01	69.59	6.06	22.3
NPCu@SiO_2_ (calcined)	7.6	0.17	12.94	27.37	59.52	–	76.1
SiO_2_@Cu	7.6	0.40	17.26	30.94	51.41	–	43.2

a n.a., not analyzed.

**Table 2:** Binding Energies and Surface Distribution of Cu Species over Cu–SiO_2_ Nanocatalysts

	component binding energy (eV) and composition (%)					
	Cu 2p_3/2_			Cu 2p_1/2_		
nanocatalyst	Cu^0^/Cu^+^	Cu^2+^	satellite	Cu^0^/Cu^+^	Cu^2+^	satellite
Cu/SiO_2_	–	934.7 (43.46)	943.6 (25.33)	–	954.6 (21.32)	962.5 (9.89)
NPCu/SiO_2_	–	934.5 (42.15)	943.4 (25.42)	–	954.4 (21.07)	960.8 (11.35)
NPCu@SiO_2_	932.6 (18.89)	935.5 (32.42)	944.1 (18.59)	952.5 (9.27)	955.2 (15.91)	962.9 (4.91)
NPCu@SiO_2_ (calcined)	933.6 (17.06)	935.5 (33.70)	944.4 (19.32)	953.5 (8.37)	955.4 (16.53)	962.9 (5.03)
SiO_2_@Cu	933.1 (7.76)	935.0 (39.43)	943.4 (22.63)	953.0 (3.81)	954.9 (19.35)	963.0 (7.02)

Nondeconvoluted Cu 2p spectra (Figure 8A) present four photoelectric peaks. They can be attributed to the Cu 2p_3/2_ (940–930 eV) and Cu 2p_1/2_ (960–950 eV) doublet and their shakeup satellites (950–940 and 970–960 eV, respectively).^57^ Fitting procedures showed that all nanocatalysts present Cu species with binding energies (BEs) between 934.5 and 935.5 eV (Cu 2p_3/2_). According to the literature,^58^ the appearance of shakeup satellites in Cu 2p spectra indicates the presence of Cu^2+^. Therefore, the component with higher BE in the Cu 2p_3/2_ region can be attributed to such a Cu^2+^ species. It is worth pointing out that there is a variation in this component BE among the studied samples (Table 2). In general, the shift to higher BE indicates that the species is more positively charged. Hence, this difference in Cu^2+^ BE suggests that the Cu–SiO_2_ interaction, as the Cu–O–Si species,^57^ is different for each catalyst, being stronger on nanocatalysts with more complex core–shell architecture as previously suggested by HRTEM investigation.

NPCu@SiO_2_ and SiO_2_@Cu Cu 2p spectra also present an additional species with BE values (932.6–933.6 eV) lower than that of Cu^2+^. Once Cu^+^ and Cu^0^ have similar BEs in the Cu 2p region,^58^ this additional component is attributed to “reduced Cu species”, i.e., Cu^+^ and/or Cu^0^. The component distribution (Table 2) indicates that the fraction of reduced Cu rises in the order Cu/SiO_2_ = NPCu/SiO_2_ (0%) < SiO_2_@Cu (11.6%) < NPCu@SiO_2_ (25.4%). Moreover, the BE of NPCu@SiO_2_ is 0.5 eV higher than the SiO_2_@Cu one, suggesting that reduced Cu is more positively charged in the former. Those results are in agreement with XANES analysis for these two samples (Figure 7). Some differences are also observed between NPCu@SiO_2_ before and after calcination (Table 2 and Figure S7). First, the amount of reduced Cu is slightly higher before calcination (28.1%), which is in agreement with XANES. Therefore, one could say that CTAB and PVP removal by calcination had just a slight effect on surface Cu oxidation. Indeed, XRD data showed that just calcined NPCu@SiO_2_ does not present CuO diffraction peaks (Figure 1). However, the reduced Cu BE increased by 1.0 eV after calcination. This shift is indicative of either an increase in the Cu oxidation state (e.g., Cu^0^ → Cu^+^) or at least a positive charging to a Cu^δ+^ state (0 < δ^+^ < 2) due to Cu partial oxidation.

The analyses of the Cu Auger LMM region (Figure 8b) can be used together with Cu 2p data to better understand the Cu species. Cu/SiO_2_ and NPCu/SiO_2_ spectra present no defined peak in this region, while a peak with a kinetic energy (KE) of ca. 915 eV is observed in NPCu@SiO_2_ and SiO_2_@Cu spectra. Considering that Cu^2+^ is observed in all analyzed catalysts and that reduced Cu was not detected on Cu/SiO_2_ or on NPCu/SiO_2_, this peak in the Cu LMM region can be associated with the presence of reduced Cu. Moreover, NPCu@SiO_2_ (Figure S7B) presents a small peak with a KE of ca. 919 eV and the one at ca. 915 eV is almost not detected. According to the literature,^60^ Cu^0^ and Cu^+^ can be distinguished in the Cu LMM region due to the difference of their KEs (ca. 3 eV). Indeed, Fan et al.^61^ have recently evaluated CuSiO_3_ reduction by *in situ* XPS, monitoring the Cu LMM region, and observed a peak with a KE of ca. 916 eV in the as-prepared sample and the appearance of a peak with a KE of ca. 919 eV after reduction. According to the authors, XPS and XANES data showed that the Cu oxidation state is between Cu^0^ and Cu^2+^ and the reduced catalyst is a material described as Cu^0^/Cu-doped SiO_2_. Hence, it can be said that reduced Cu in the presented nanocatalysts is in the form of an oxidation state Cu^δ+^ (0 < δ < 2) and that the peak at the KE of ca. 915 eV indicates an oxidation state more similar to Cu^+^ while the peak at the KE of ca. 919 eV can be associated with a more “Cu^0^-like” oxidation state.

Taking into consideration these XPS data along with the previously discussed bulk characterization analyses, it can be concluded that the absence of reduced Cu on the Cu/SiO_2_ ordinary catalyst is in accordance with XRD and H_2_-TPR data. This catalyst showed well-defined CuO phase peaks in its diffractogram (Figure 1A) and a clear peak in the reduction profile attributed to CuO reduction to Cu^0^ (Figure 6). As for NPCu@SiO_2_, XPS data showed that surface Cu is quite sensible to oxidation and keeping CTAB and PVP after NPCu@SiO_2_ synthesis is useful for preventing oxidation. Although the diffractogram of just calcined NPCu@SiO_2_ showed no CuO peaks (Figure 1), XPS indicated that its surface is at least partially oxidized. Moreover, Cu^2+^ is already present on this catalyst surface before calcination. NPCu synthesis is expected to generate Cu^0^ nanoparticles recovered by PVP. However, NPCu washing is performed before their use in NPCu@SiO_2_ synthesis to remove the excess of PVP. It is conceivable that such washing promoted NPCu partial surface oxidation before encapsulation, explaining the presence of Cu^2+^ on the NPCu@SiO_2_ surface. Finally, SiO_2_@Cu presented no Cu-phase diffraction peaks in its diffractogram (Figure 1A) but its H_2_-TPR profile showed two reduction peaks (Figure 6). XPS data showed that the SiO_2_@Cu surface is mainly composed of Cu^2+^ but also presents reduced Cu (Cu^δ+^, where 0 < δ < 2). Hence, the absence of Cu-phase diffraction peaks can be attributed to the presence of small Cu crystal domains, which can be related to a stronger Cu–SiO_2_ interaction in this sample, relative to Cu/SiO_2_.

**Figure 9:**
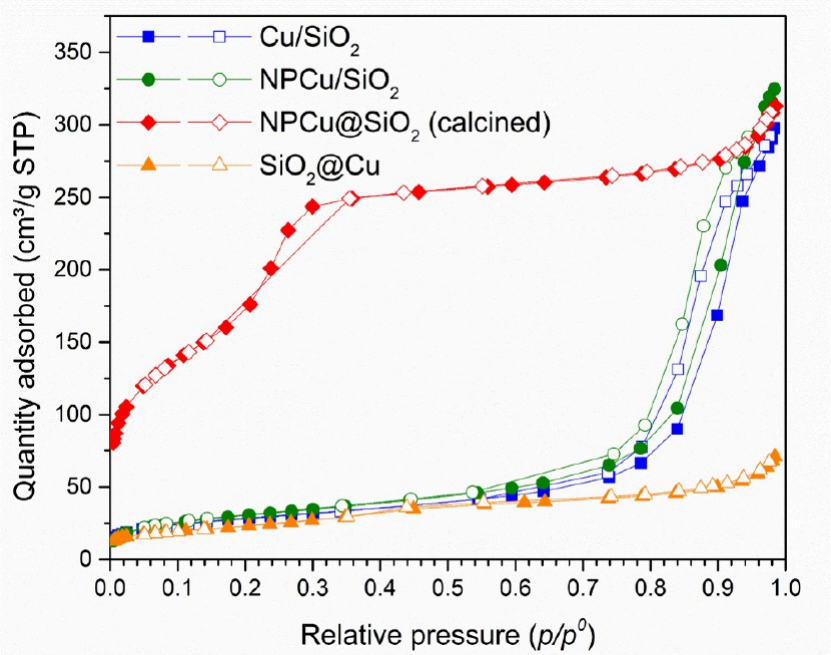
N_2_ physisorption isotherms of the synthesized nanocatalysts.

The nanocatalysts’ textural properties obtained by nitrogen physisorption are presented in Figure 9 and Table 3. Comparing the isotherms, it can be seen that all of them present an inflection point at low *p*/*p*^0^ values; Cu/SiO_2_ and NPCu/SiO_2_ ones present a second inflection point at high *p*/*p*^0^ values, while the adsorbed quantity on SiO_2_@Cu and NPCu@SiO_2_ continually increases until *p*/*p*^0^ = 1. Finally, Cu/SiO_2_ and NPCu/SiO_2_ present clear hysteresis loops which occur in a narrow range of high *p*/*p*^0^ values and a wide range of N_2_ adsorbed quantity. According to IUPAC classification,^62^ Cu/SiO_2_ and NPCu/SiO_2_ isotherms are type IVa, characteristic of mesoporous materials, with H1 hysteresis loops, indicating the presence of ink-bottle pores. SiO_2_@Cu and NPCu@SiO_2_ isotherms, on the other hand, are type II isotherms, typical of nonporous or macroporous materials.

**Table 3:** BET Surface Areas, Pore Volumes, and Average Pore Diameters of Cu–SiO_2_ Nanocatalysts

nanocatalyst	BET surf. area (m^2^ g^–1^)	pore vol^a^ (cm^3^ g^–1^)	pore diam^a^ (Å)
Cu/SiO_2_	101	0.461	141
NPCu/SiO_2_	110	0.503	132
NPCu@SiO_2_	797	0.491	24
SiO_2_@Cu	84	0.111	45

a Estimated from BJH desorption branch.

The calculated textural properties of Cu/SiO_2_ and NPCu/SiO_2_ are quite similar as summarized in Table 3. Once both catalysts were prepared by Cu impregnation/deposition over the same SiO_2_ support, it can be said that those results are mainly attributed to SiO_2_ textural properties. SiO_2_@Cu presented lower BET surface area, pore volume, and average pore diameter, which is ascribed to the intrinsic features of the nonporous SiO_2_ spheres initially synthesized by the Stöber method. Finally, NPCu@SiO_2_ presents a significantly higher BET surface area value with pore volume and average diameter similar to those of the supported catalysts and SiO_2_@Cu, respectively. The synthesis of this nanocatalyst is based on TEOS polymerization around NPCu, forming a SiO_2_ shell. In this procedure, the addition of CTAB promotes the formation of a pore network and, therefore, the nanocatalyst final textural properties are expected to be more developed.

**Scheme 1:**
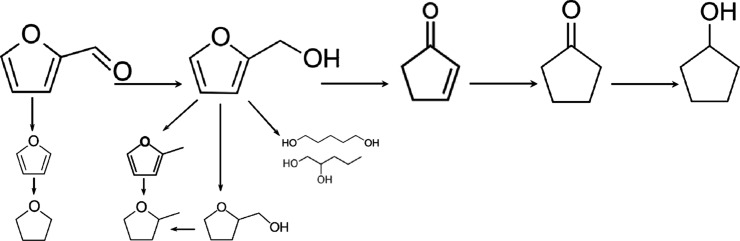
Simplified Reaction Network of Furfural Hydrogenation over Metal Catalysts

Activities of all Cu-based nanocatalysts were assessed in the aqueous-phase hydrogenation of furfural at 150 °C and 30 bar hydrogen pressure and initially performed at the same furfural:Cu molar ratio. The reaction network of furfural hydrogenation is quite complex with a multitude of possible products due to the multifunctional nature of furfural, which holds a carbonyl (C=O) group and π-conjugated C=C bonds in a five-membered-ring backbone. Therefore, hydrogenated compounds such as furfuryl alcohol, tetrahydrofurfuryl alcohol, 2-methylfuran, and 2-methyltetrahydrofuran can be obtained. Other compounds can also be produced via cascade reactions coupled to hydrogenation, such as 2-cyclopenten-1-one and cyclopentanone (hydrogenation–rearrangement), cyclopentanol (hydrogenation–rearrangement–hydrogenation), and pentanediols (hydrogenation–ring opening). Finally, side reactions may lead to furan (decarbonylation) and tetrahydrofuran (decarbonylation–hydrogenation). All these reaction pathways are illustrated in Scheme 1. Catalyst composition (nature of metal phase and support), nature of the surface sites, and nature of the solvent used in the reaction have been claimed as the main factors driving product distribution.^4,5,7,63^

Considering the Cu-based nanocatalysts investigated in this study, both catalytic activity and product distribution were distinctively different (Table 4) despite all samples holding the same chemical composition, i.e., the same metal phase and support. It is worth mentioning that a control experiment ran without catalyst revealed that no substantial furfural conversion can be accomplished (Table 4, entry 1). Therefore, the kinetic data in Table 4 express the behaviors of the nanocatalysts and clearly evidence the impact of their architectures on performance. Nonetheless, catalytic performance cannot be associated with the physical properties of the nanocatalysts derived from their architectures as the highest furfural conversion was reached over the solid with the lowest BET surface area and pore volume (SiO_2_@Cu) as summarized in Table 3. Furthermore, it cannot either be related to the number of metallic sites on each nanocatalyst (Cu/SiO_2_ = 4.0 mmol/g_cat_; NPCu/SiO_2_ = 1.8 mmol/g_cat_; NPCu@SiO_2_ = 1.5 mmol/g_cat_; SiO_2_@Cu = 1.2 mmol/g_cat_) as estimated from their mean particle sizes determined from TEM examination. It can thus be inferred that the architecture of the nanocatalyst tailors more refined chemical characteristics that rule catalytic behavior, which could indeed be predicted in view of the characterization study discussed earlier. In relation to the nanocatalyst chemical stabilities, ICP data indicated that Cu leaching remained below 2% after 4 h of furfural aqueous phase hydrogenation at 150 °C and 30 bar (H_2_) (Table 4, entries 2–5). Therefore, all catalysts were resistant against leaching under the tested conditions.

**Table 4:**
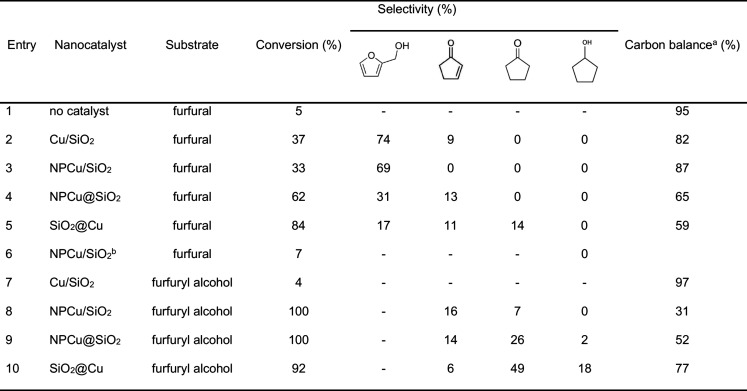
Performances of All Nanocatalysts: Substrate Conversion, Product Selectivity, and Carbon Balance^c^

a As determined by HPLC analyses.

b Not reduced before reaction.

c Reaction conditions: 150 °C, 30 bar H_2_, substrate:Cu mass ratio 5:1, 4 h reaction.

Conventional Cu/SiO_2_ catalyst led basically to the formation of furfuryl alcohol (Table 4, entry 2), evidencing the well-known selectivity of copper to preferentially reduce C=O groups over C=C bonds in the furan ring.^64^ Its performance is comparable to those reported in the literature for analogous Cu/SiO_2_ catalysts under similar reaction conditions.^29^ The NPCu/SiO_2_ nanocatalyst presented a very similar behavior (Table 4, entry 3), revealing that deposition of previously synthesized copper nanoparticles onto the silica surface allows a more metal particle size controlled catalyst but with no significant impact on product distribution in the aqueous-phase hydrogenation of furfural. It is likely due to the negligible influence of metal particle size on furfural adsorption, which is reckoned to be the η^1^-(*O*)-aldehyde tilted configuration.^41,65−67^ In this case, furfuryl alcohol is formed through C=O reduction while preserving the C=C bonds as the furan ring is not bound to the metal surface.^41^ Furthermore, the catalytic behavior of NPCu/SiO_2_ corroborates the previous conclusion that such a preparation protocol renders a weak interaction between active Cu nanoparticles and the SiO_2_ support. Indeed, preparation of well-defined size-controlled metal nanoparticles and their subsequent deposition/immobilization on a support has been credited with excluding any influence of the support from the catalyst performance.^68−70^

Encapsulation of NPCu into silica did lead to a distinct catalytic performance in furfural hydrogenation as both furfuryl alcohol and 2-cyclopenten-1-one were formed over the NPCu@SiO_2_ sample (Table 4, entry 4). A similar behavior was accomplished over the SiO_2_@Cu nanocatalyst; however, the cascade process proceeded even further as cyclopentanone was also produced (Table 4, entry 5). The somewhat better performance of SiO_2_@Cu could be associated with the higher surface metal concentration as revealed by XPS (Table 1). These results unveiled that a rearrangement second cascade step (Scheme 1) is promoted as well, evidencing that metallic Cu^0^ centers are not the sole active sites and thus an additional active surface site coexists. Rearrangement of furfuryl alcohol indeed demands surface acid sites as it proceeds as an initial acid-mediated dehydration and ring opening followed by a rearrangement of the carbacycle.^71^ These results, therefore, substantiate the greater interaction between Cu and SiO_2_ as the Cu–O–Si species accomplished through synthesizing those core–shell nanostructures as well as the occurrence of oxidic Cu^δ+^ species as disclosed by bulk and surface characterization results discussed hereinbefore. Such species are indeed Lewis acid sites and are proposed to act alongside Cu^0^ centers to promote the cascade formation of cycloketones in core–shell nanocatalysts.^72^ The interaction of oxidic Cu^δ+^ species and SiO_2_ has recently been claimed as the only source of acid sites in Cu/SiO_2_ catalysts, and they have been shown to play an effective role in catalyzing glycerol dehydration to hydroxyacetone.^59^ Additionally, the role played by Cu^δ+^ species as Lewis acid sites to polarize the C=O bond^21,72,73^ in the furfural molecule in the η^1^-(*O*)-aldehyde mode has also been appealed.^74^ Finally, it should be emphasized that, even though cyclopentanone formation from furfuryl alcohol proceeds through 4-hydroxycyclopent-2-enone, 3-hydroxycyclopentanone, and cyclopent-2-enone,^71,75^ the hydroxycycloketones were not detected in these experiments, suggesting those intermediates are easily hydrogenated under the conditions applied herein. A similar trend has indeed been reported recently in the literature.^21,25,76,77^ Low carbon balances achieved in some reactions may be attributed to the high reactivity of furfural and especially that of furfuryl alcohol, which is the major product in these reactions and the first one to be formed in the reaction network leading to cycloketones. It would lead to side reactions such as resinification and polymerization rendering high-molecular-weight compounds and humins.^13,78^ Indeed, furfuryl alcohol polymerization is thermodynamically favored in water and acidic medium,^78^ which are conditions fulfilled in the present study.

A complementary test was carried out over an unreduced as-synthesized NPCu@SiO_2_ nanocatalyst, and the result is also collected in Table 4, entry 6. It should be recalled that this as-synthesized sample still holds a fraction of organic compounds used to stabilize and allow size control of metal nanoparticles (PVP) and to generate a porous network during silica formation through TEOS hydrolysis (CTAB). The presence of such agents was indeed evidenced by TG (Figure 2) and XPS analyses (Table 1). This sample was not active at all, leading to a result similar to the blank experiment ran without any catalyst (Table 4, entry 1). This result reveals that furfural hydrogenation over NPCu is inhibited by the remaining stabilizing agent capping the nanoparticle and hindering the accessibility of substrates (furfural and hydrogen) to the metal surface. Therefore, the prior stabilizer removal reaction is crucial in this case as it is the way to obtain an active core–shell nanocatalyst.

Comparing the performances of different Cu/SiO_2_ catalysts with other reports in the literature regarding furfural aqueous-phase hydrogenation is not a simple and straightforward task as different catalyst architectures, which are mostly SiO_2_-supported Cu catalysts prepared through different protocols holding different copper contents,^29,41,67^ and distinct reaction conditions (temperature, hydrogen pressure, reaction time, and furfural:Cu ratio) are usually applied. Still, the performances of the catalysts studied in this present work are contrasted with those from the literature in Table S1.

Formation of cycloketones via a cascade reaction step from furfuryl alcohol over Cu^δ+^ acidic centers was assessed by using furfuryl alcohol as the substrate instead of furfural, and the results are also summarized in Table 4 (entries 7–10). It is seen that the ordinary Cu/SiO_2_ catalyst is not active for furfuryl alcohol conversion in line with its inaptness to hydrogenate C=C bonds. Additionally, it consistently evidences that this catalyst does not possess the active oxidic Cu^δ+^ centers to promote furfuryl alcohol rearrangement.

Contrarily, all other nanocatalysts led to the formation of cycloketones and, in the case of core–shell catalysts, also cyclopentanol. Once again, no hydrocycloketones were detected as intermediates. It is worth noting that furfuryl alcohol transformation goes further to rearranged–hydrogenated products (Scheme 1) as the interaction between Cu and silica is strengthened in core–shell architecture. The performance of SiO_2_@Cu (Table 4, entry 10) stands out as the formation of cyclopentanone and cyclopentanol sums around 70% of products at a carbon balance of 77%. Even lower carbon balances calculated over other samples (Table 4, entries 8 and 9) are also a consequence of the significant formation of some unidentified compounds.

**Figure 10:**
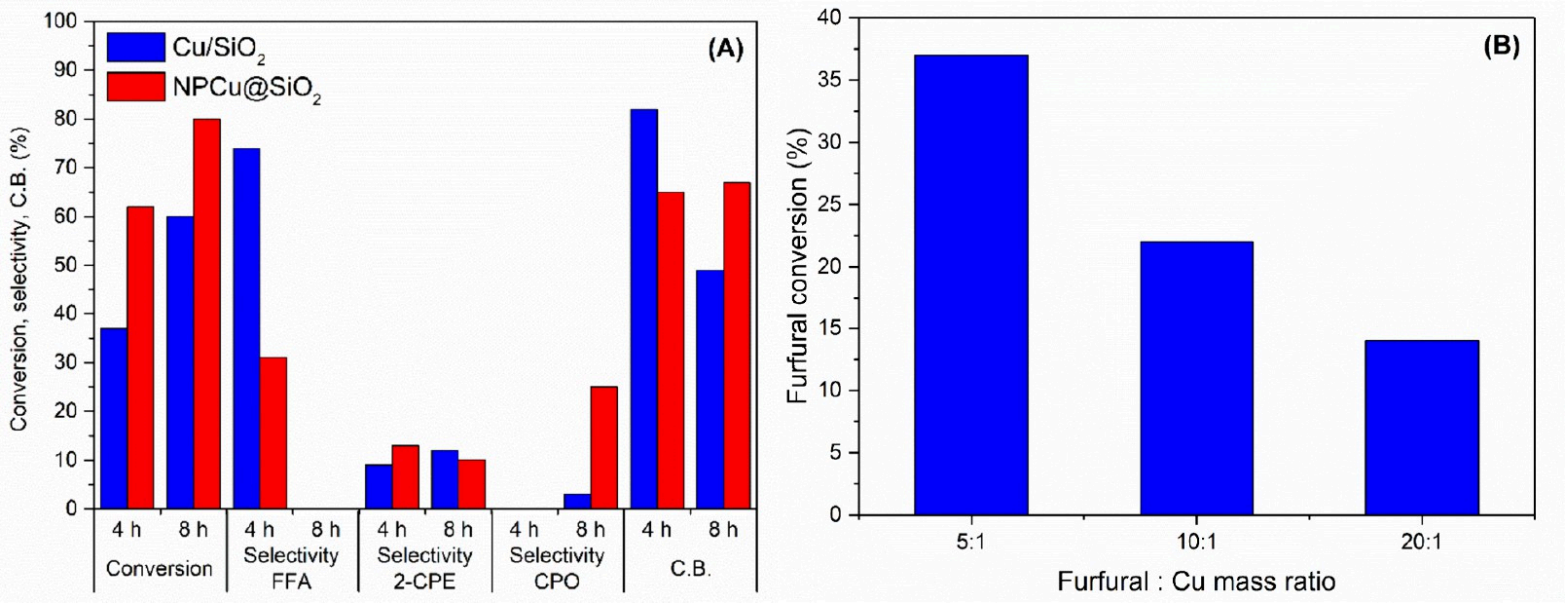
(A) Furfural conversion; selectivity to furfuryl alcohol (FFA), 2-cyclopenten-1-one (2-CPE), and cyclopentanone (CPO); and carbon balance (CB) over Cu/SiO_2_ and NPCu@SiO_2_ catalysts after 4 and 8 h reactions. (B) Furfural conversion over Cu/SiO_2_ at different furfural:Cu molar ratios. Reaction conditions: 150 °C, 30 bar H_2_.

Additional runs were carried out with selected catalysts to evaluate their performances under broader reaction conditions, and the results are collected in Figure 10. Increasing the reaction time up to 8 h leads to an expected increase in conversion and renders a very different product distribution pattern (Figure 10A) as furfuryl alcohol is no longer the major product. Its disappearance is suggested to occur due to furfuryl alcohol polymerization, which is known to be thermodynamically favored in aqueous solution.^79^ It is indeed corroborated by the lower carbon balance reached after 8 h of reaction as determined by HPLC analyses. Conversely, pushing up the furfuryl alcohol initial concentration provokes a drastic drop in conversion (Figure 10B). These data altogether indicate that working at high furfuryl alcohol concentration or at longer reaction time frame should be avoided as losing carbon by the formation of heavy compounds, mostly probably from polymerization and condensation reactions, is prone to occur.

These results disclose that performing furfural hydrogenation in water is not the sole condition to produce cycloketones as furfuryl alcohol is the major product once catalysts with weak or no Cu–support interaction (Cu/SiO_2_ and NPCu/SiO_2_) are used. The establishment of such interactions, which allows the formation of electron-deficient Cu^δ+^ moieties, particularly Cu^2+^ and Cu^+^ as unveiled by XPS and XANES investigations, is critical to promoting the hydrogenation–ring rearrangement cascade mechanism leading to cycloketones. It was shown that Cu^δ+^–SiO_2_ interactions can be tuned by simply designing more advanced nanoarchitectures, particularly core–shell nanocatalysts. Finally, it is tentatively concluded that the electron-deficient nature of the metal–support interface species does not need to be strong to play a role in the cascade mechanism and promote the demanded dehydration step during furfuryl alcohol rearrangement to cycloketones.

As discussed hereinbefore, the chemical stabilities of the synthesized catalysts are good as they revealed to be resistant against leaching under the tested conditions. Structural and morphological changes were also assessed by collecting and analyzing the nanocatalysts after reaction, and the results are displayed in Figures S8 and S9.

The diffractograms of the spent nanocatalysts (Figure S8) showed differences in the crystalline phases present. Diffraction peaks related to Cu^0^ (COD 9013014) and Cu_2_O (COD 1000063) phases are now observed in Cu/SiO_2_, NPCu/SiO_2_, and SiO_2_@Cu samples, while only an unresolved peak is observed at 36.6° for NPCu@SiO_2_, which is attributed to the (111) plane of Cu_2_O. It is worth noting that Cu/SiO_2_ was the only one exhibiting well-resolved Cu-phase diffraction peaks before activation and reaction, but only a crystalline CuO phase was present. The occurrence of Cu^0^ and Cu_2_O in the spent catalyst (Figure S8) is clearly a consequence of the catalyst reduction prior to furfural hydrogenation. As for the others, reduced Cu^0^ phase would be expected from the beginning as already discussed and experimentally evidenced hereinbefore. The clear detection of both Cu^0^ and Cu_2_O phases in the spent samples can be taken as evidence that Cu particle agglomeration may take place during the aqueous-phase reaction, particularly for NPCu/SiO_2_ and SiO_2_@Cu nanocatalysts. On the other hand, these XRD patterns indicate that NPCu encapsulation by SiO_2_ in NPCu@SiO_2_ is effective in improving thermal stability and avoiding Cu nanoparticle clustering. These XRD findings are indeed confirmed by complementary TEM examination of the spent nanocatalysts (Figure S9) as no significant changes are observed between fresh and spent NPCu@SiO_2_ (Figure S9a) while clear agglomeration is noted for NPCu/SiO_2_ (Figure S9b). The SiO_2_@Cu nanocatalyst also shows NPCu agglomeration as depicted in Figure S9c. Additionally, the collapse at some extent of these SiO_2_@Cu nanostructures can be noticed (Figure S9d). EDX mapping indeed discloses the occurrence of both dispersed and agglomerated NPCu in SiO_2_@Cu after reaction (Figure S9c).

Altogether, the characterization of the Cu–SiO_2_ nanocatalysts after furfural aqueous-phase hydrogenation indicates that the creation of a deeper and stronger Cu–SiO_2_ interaction through catalyst nanoarchitecturing may not be enough to ensure morphological stability. Therefore, despite the nanocatalyst activities and higher selectivities toward cycloketones, their long-term performances may be affected by morphological changes in water.

## Conclusions

Cu–SiO_2_ nanocatalysts with distinct architectures were successfully synthesized, and different interactions between Cu and SiO_2_ could be effectively established. The degree of intimacy between them was shown to directly impact catalytic activity and, more importantly, selectivity. Supporting/depositing copper nanoparticles onto silica leads to weak Cu–SiO_2_ interactions, and hydrogenation of the C=O bond to furfuryl alcohol is the main reaction pathway even in water. It was unveiled that water is not the only condition required to promote the hydrogenation–ring rearrangement cascade reaction from furfural to cycloketones. On the other hand, encapsulation techniques to synthesize both NPCu@SiO_2_ and SiO_2_@Cu core–shell nanostructures allowed the coexistence of Cu^0^ and electron-deficient Cu^δ+^ moieties in strong interaction with SiO_2_, particularly Cu^2+^ and Cu^+^ as unveiled by spectroscopic investigations, which were found to drive product distribution to cycloketones. It was proposed that Cu^0^ and oxidic Cu^δ+^ species work cooperatively to promote furfural adsorption, hydrogen dissociative chemisorption, and reaction intermediate dehydration. Despite their potential catalytic performances, morphological changes were promoted for SiO_2_@Cu while NPCu@SiO_2_ was revealed to be very stable with no metal agglomeration during reaction in water.

## Supporting Information Available

Relative mass derivatives of NPCu/SiO_2_, NPCu@SiO_2_, CTAB, and PVP, TEM and STEM micrographs and particle size distributions, TEM and STEM-in-SEM micrographs of NPCu, STEM-HAADF, TEM micrographs and EDX elemental mapping of NPCu@SiO_2_, TEM micrographs and EDX elemental mapping of NPCu@SiO_2_ catalyst after calcination, TEM micrographs of ultramicrotome sliced NPCu@SiO_2_, XPS spectra of NPCu@SiO_2_ before and after calcination, diffractograms of Cu–SiO_2_ after furfural hydrogenation, TEM micrographs and EDX elemental mapping of NPCu@SiO_2_, NPCu/SiO_2_, and SiO_2_@Cu after furfural hydrogenation, STEM-HAADF of spent SiO_2_@Cu, comparison of catalyst performance with the literature (PDF)
