# Supplementary material for: Tailoring Cu–SiO2 Interaction through Nanocatalyst Architecture to Assemble Surface Sites for Furfural Aqueous-Phase Hydrogenation to Cycloketones
Source: ACS Appl Mater Interfaces. 2024 Jul 30;17(9):13146–61. doi: 10.1021/acsami.4c05266 (PMC11891833; doi:10.1021/acsami.4c05266)
Supplement: Supplementary file 1 — am4c05266_si_001.pdf [file am4c05266_si_001.pdf]

SUPPORTING INFORMATION

Tailoring Cu-SiO_2 interaction through nanocatayst architecture to assemble surface sites for furfural aqueous-phase hydrogenation to cycloketones

Welington L. S. Soares,[1†] Leon F. Feitosa,[2†] Carla R. Moreira,[2] Francine Bertella,[3] Christian Wittee Lopes,[3] Andréa M. Duarte de Farias,[2] Marco A. Fraga[1,2*]

[1]Instituto Militar de Engenharia, Praça Gen. Tibúrcio, 80, Urca, Rio de Janeiro, RJ, 22290-270, Brazil

[2]Instituto Nacional de Tecnologia – INT, Laboratório de Catálise, Av. Venezuela, 82/518, Saúde, Rio de Janeiro, RJ, 20081-312, Brazil

[3]Departamento de Química, Universidade Federal do Paraná (UFPR), Curitiba, PR, 81531-990, Brazil.

[†]These authors contributed equally

*corresponding author: marco.fraga@int.gov.br

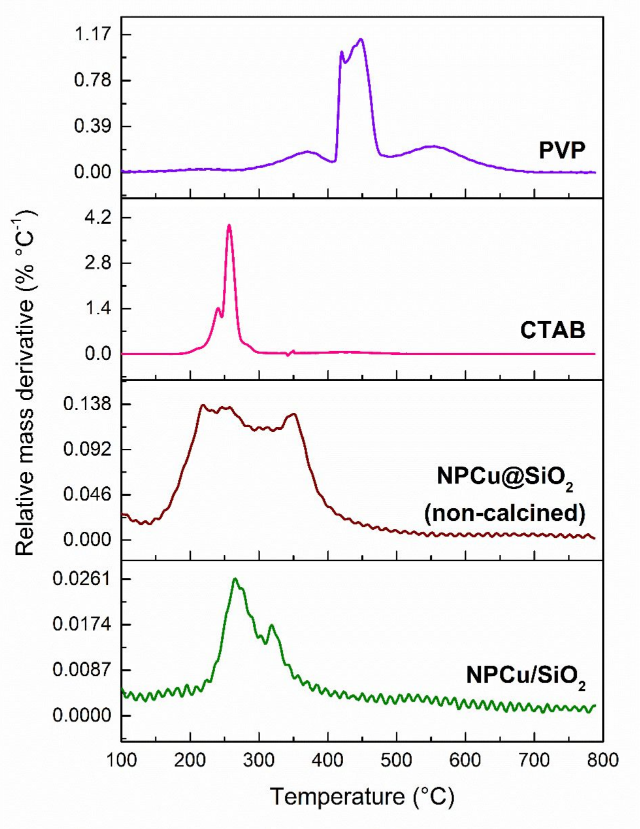

Figure S1. Relative mass derivatives of NPCu/SiO_2, NPCu@SiO_2, CTAB and PVP obtained by TGA in oxidizing atmosphere.

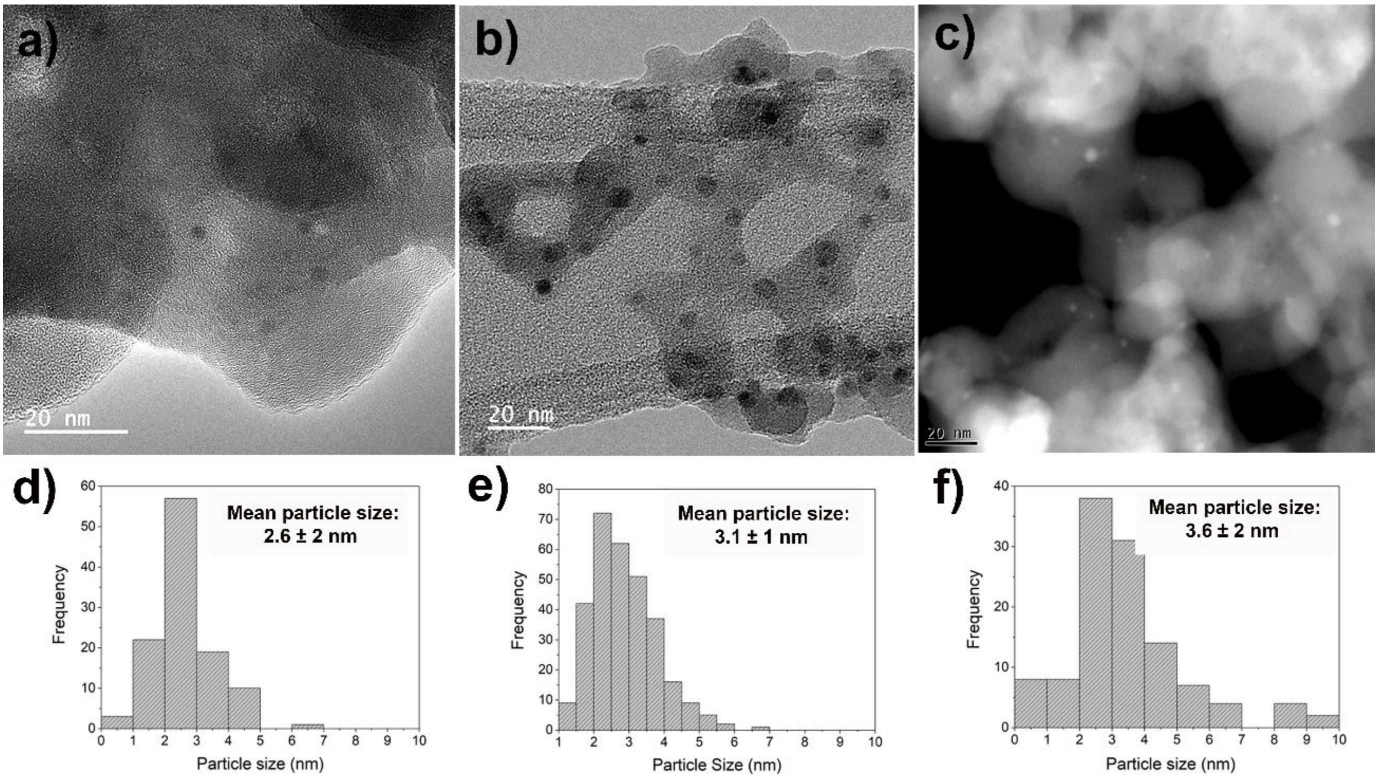

Figure S2. TEM micrographs and particle size distribution for (a,d) Cu/SiO_2 and (b,e) copper nanoparticles (NPCu), and STEM micropraph and particle size distribution for (c,f) $NPCu/SiO_2$.

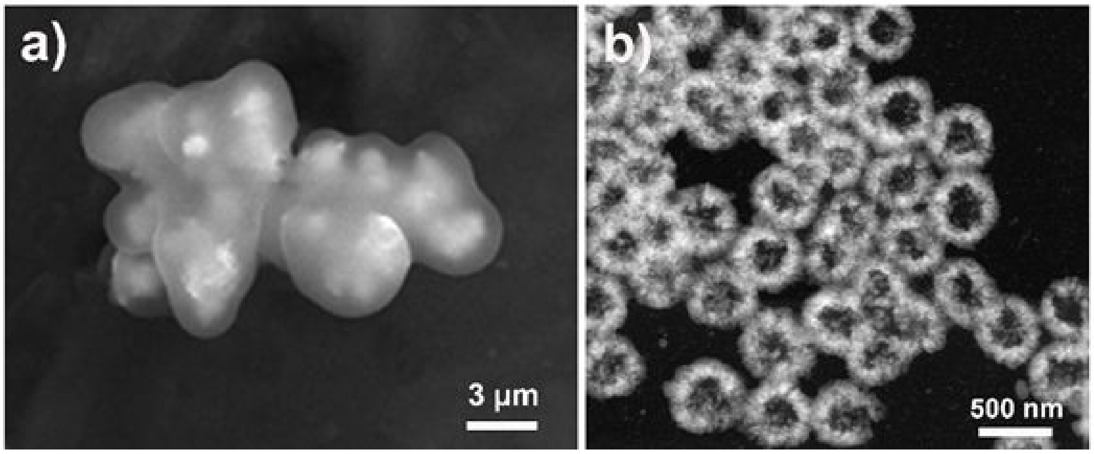

Figure S3. (a) SEM and (b) *STEM in SEM* (HAADF detector) micrographs of copper nanoparticles (NPCu).

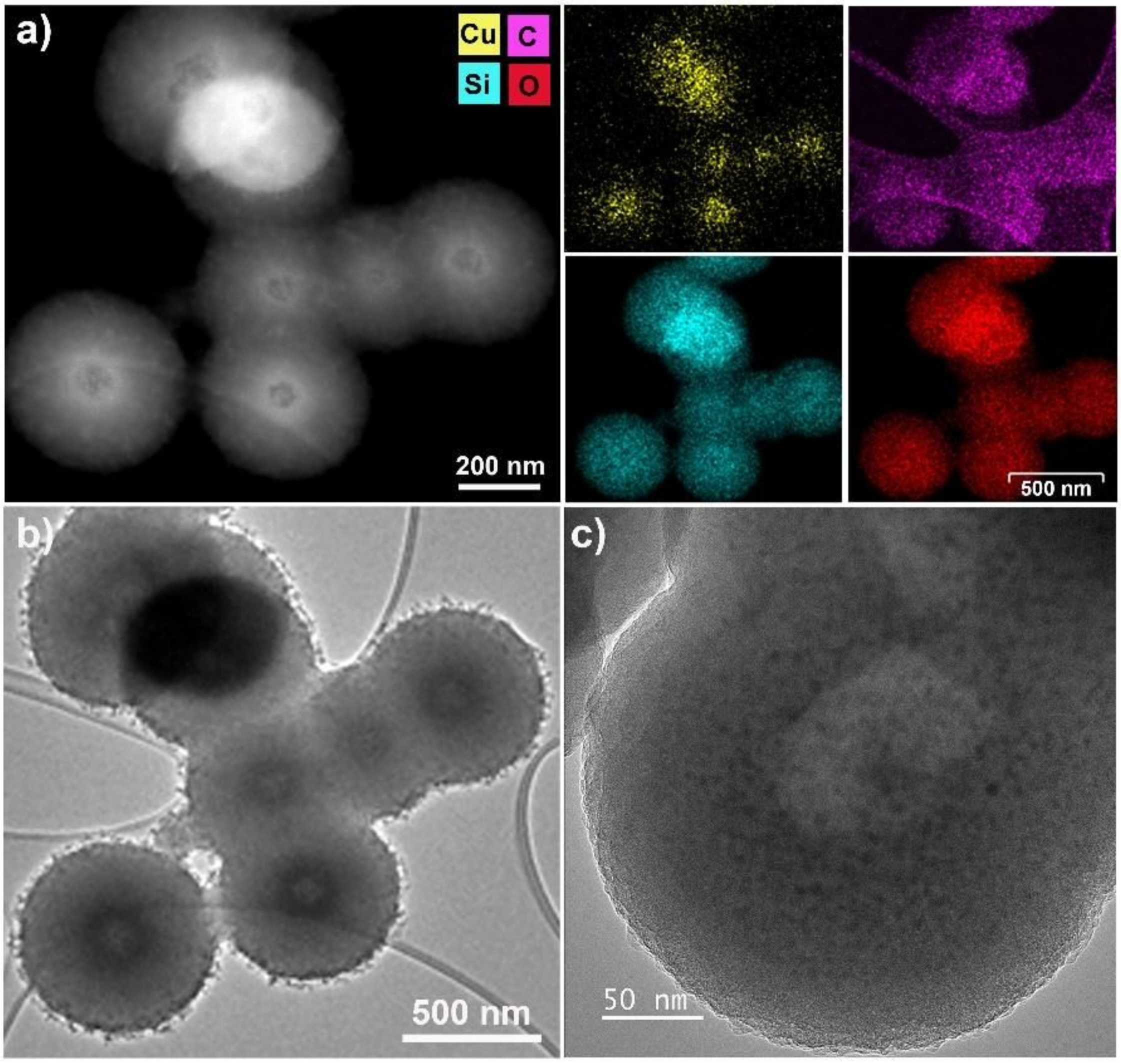

Figure S4. (a) STEM-HAADF and (b,c) TEM micrographs of NPCu@SiO_2 nanocatalyst. EDX elemental mapping of (a,b) micrographs: Si-cyan, Cu-yellow, C-magenta and O-red.

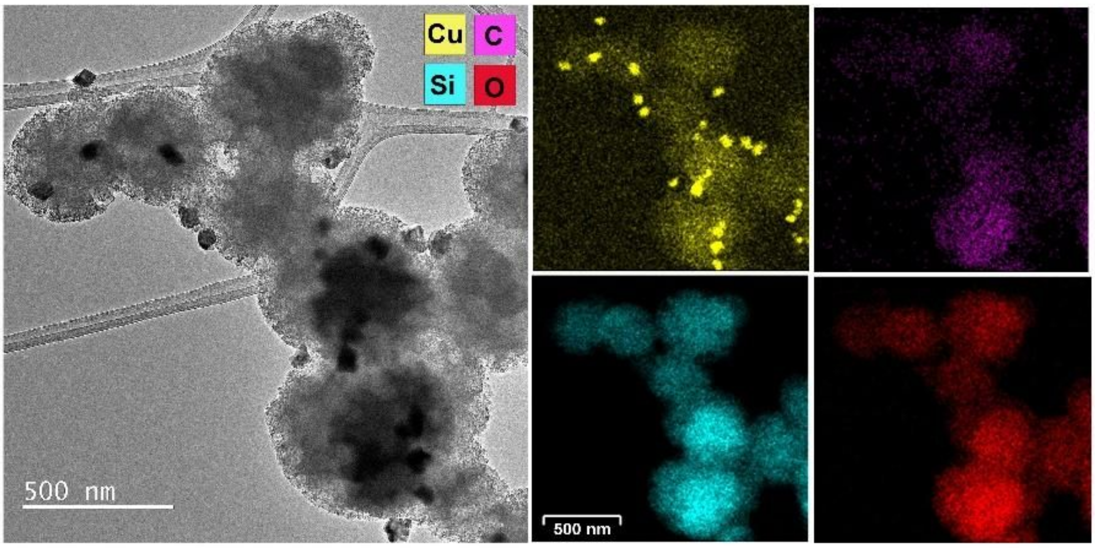

Figure S5. TEM micrographs of NPCu@SiO_2 catalyst after calcination and it EDX elemental mapping: Si-cyan, Cu-yellow, C-magenta and O-red.

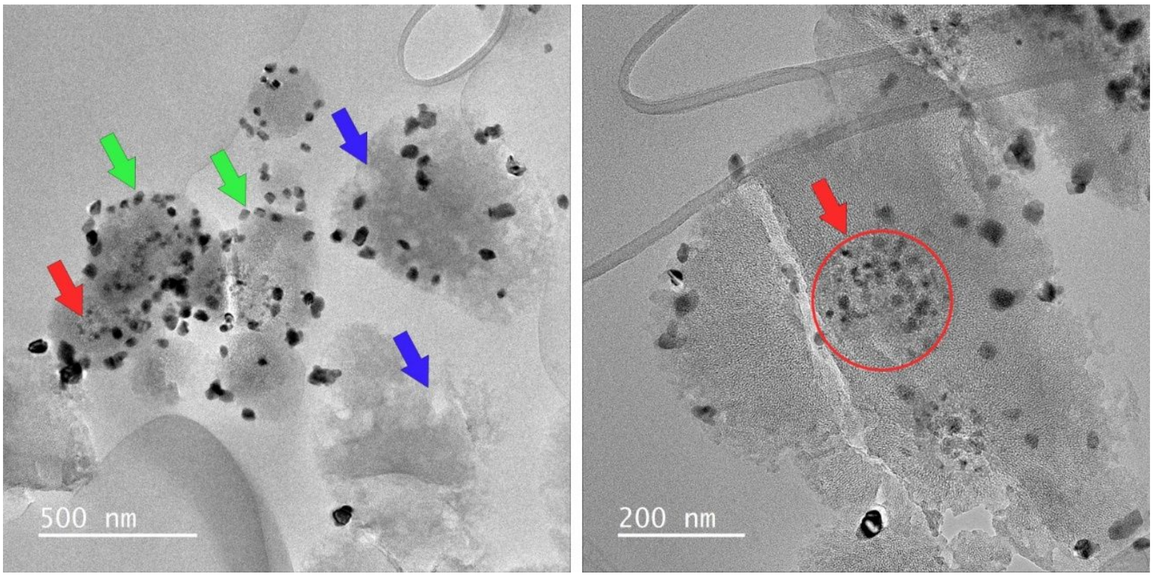

Figure S6. TEM micrographs of ultramicrotome sliced NPCu@SiO_2 catalyst after calcination. Green arrows indicate NPCu on the SiO_2 surface; red arrows point the NPCu in the core of the core-shell structure, and blue arrows show SiO_2 pores.

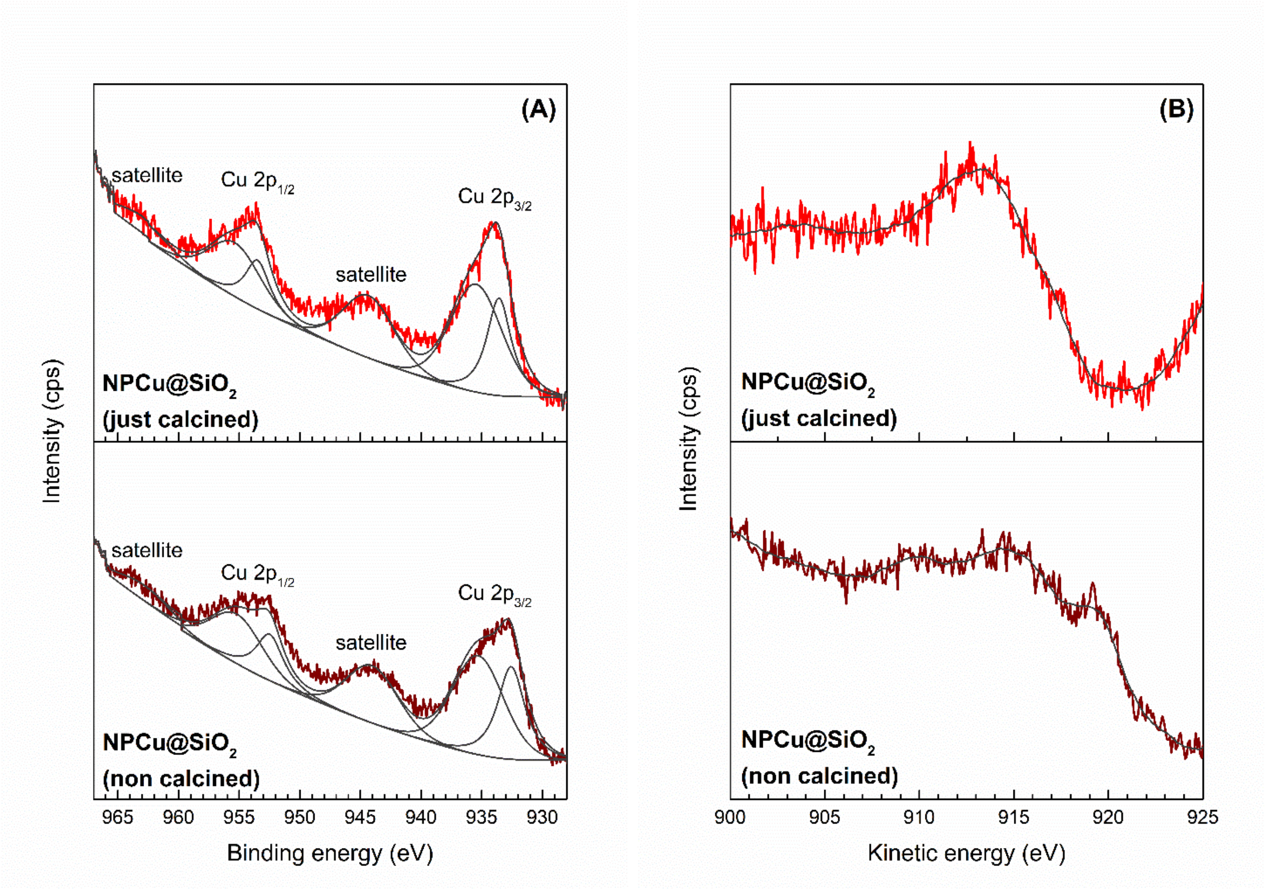

Figure S7. XPS spectra of NPCu@SiO_2 before and after calcination. (A) Cu 2p region; (B) Cu LMM (Auger) region.

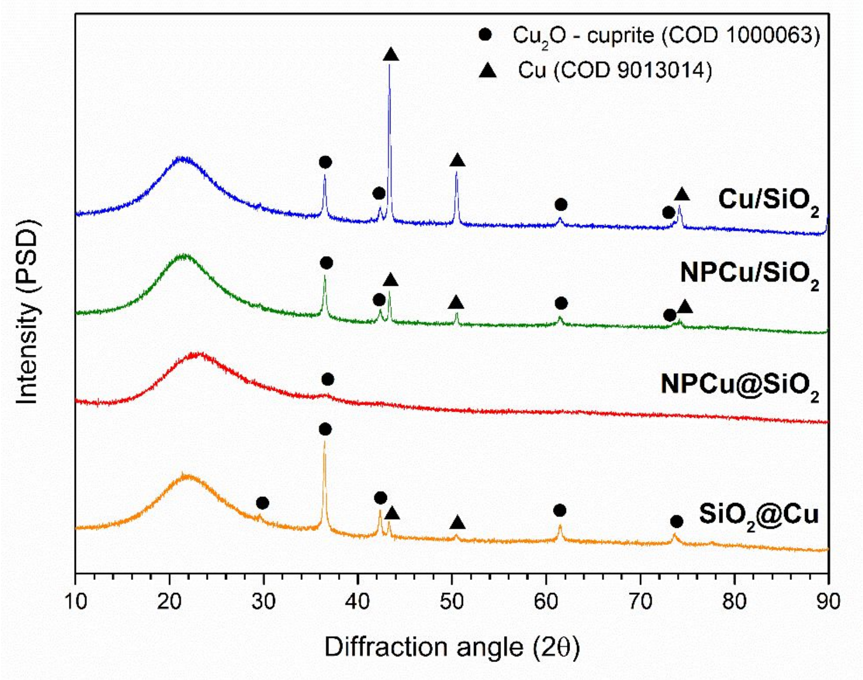

Figure S8. Diffractograms of Cu-SiO_2 nanocatalysts after furfural aqueous-phase hydrogenation.

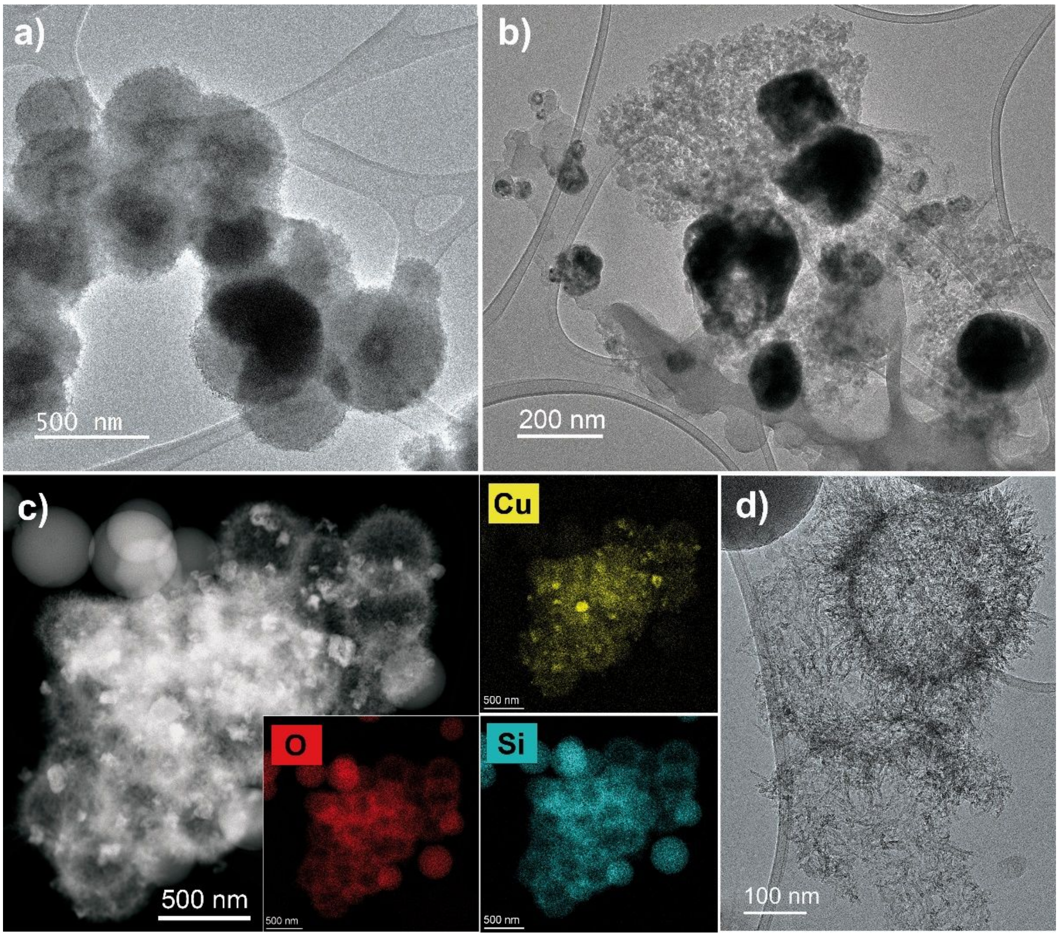

Figure S9. TEM micrographs of (a) NPCu@SiO_2, (b) NPCu/SiO_2, and (d) SiO_2@Cu nanocatalysts after furfural aqueous-phase hydrogenation. STEM-HAADF of spent SiO_2@Cu (c) and their respective EDX elemental mapping: Si-cyan, Cu-yellow and O-red.

Table S1. Comparison of catalyst performance from this work with the literature for furfural aqueous-phase hydrogenation over Cu-SiO_2 systems.

Ref.[a]	catalyst	furfural:Cu mass ratio	%Cu	T (^oC)	P_{H2} (bar)	t (h)	X (%)	S_{FFA}[b] (%)	S_{2MF}[c] (%)	$S_{cycloketones}$[d] (%)
29	Cu/SiO_2	36:1	20	140	40	8	68	9	0	90
41	Cu/SiO_2	39:1	20	120	25	4	25	58	37	0
41	Cu/SiO_2	39:1	20	120	25	4	45	71	17	0
41	Cu/SiO_2	39:1	20	120	25	4	95	68	7	0
67	Cu/SiO_2	30:1	25	150	20	6	55	31	0	61
This work	Cu/SiO_2	5:1	9.3	150	30	4	37	74	0	9
This work	NPCu/SiO_2	5:1	5.8	150	30	4	33	69	0	0
This work	NPCu@SiO_2	5:1	7.6	150	30	4	62	31	0	13
This work	SiO_2/Cu	5:1	7.6	150	30	4	84	17	0	25

[a]reference numbers relate to those listed in the manuscript

[b]selectivity to furfuryl alcohol (FFA)

[c]selectivity to 2-methylfuran (2MF)

[d]all cycloketones produced were grouped
